# A homozygous CTLA-4 variant causes CTLA-4 deficiency with severe immune dysregulation

**DOI:** 10.70962/jhi.20250227

**Published:** 2026-06-09

**Authors:** Mehmet Cihangir Catak, Salim Can, Satanay Hubrack, Feyza Bayram Catak, Asha Elmi, Royala Babayeva, Razin Amirov, Melek Yorgun Altunbas, Sevgi Bilgic Eltan, Deniz Ertem, Baris Yilmaz, Ahmet Koc, Batu Erman, Emine Bozkurtlar, Elif Karakoc-Aydiner, Ahmet Ozen, Bernice Lo, Safa Baris

**Affiliations:** 1 Department of Pediatric Allergy and Immunology, Faculty of Medicine, Marmara University, Istanbul, Turkey; 2 Istanbul Jeffrey Modell Diagnostic and Research Center for Primary Immunodeficiencies, Istanbul, Turkey; 3 The Isil Berat Barlan Center for Translational Medicine, Istanbul, Turkey; 4 Division of Translational Medicine, Research Branch, Sidra Medicine, Doha, Qatar; 5 Division of Pediatric Gastroenterology Hepatology and Nutrition, Department of Pediatrics, Faculty of Medicine, Marmara University, Istanbul, Turkey; 6 Department of Pediatric Hematology Oncology, Faculty of Medicine, Marmara University, Istanbul, Turkey; 7 Department of Molecular Biology and Genetics, Faculty of Engineering and Natural Sciences, Acıbadem University, İstanbul, Turkey; 8 Department of Pathology, Faculty of Medicine, Marmara University, Istanbul, Turkey; 9 College of Health and Life Sciences, Hamad Bin Khalifa University, Doha, Qatar

## Abstract

CTLA-4 is a critical immune checkpoint that maintains self-tolerance by regulating immune activation. Here, we describe the first case of homozygous CTLA-4 deficiency (*CTLA4*^*S172P/S172P*^), presenting with early-onset autoimmunity, lymphoproliferation, and growth failure. Immunological profiling revealed profound T- and B-cell dysregulation, characterized by T-cell hyperproliferation, a T_H_1-skewed helper T-cell phenotype, and expansion of activated and atypical B-cell subsets. The identified variant led to impaired CTLA-4 protein stability and enhanced lysosomal degradation, resulting in significantly reduced but still detectable total and surface expression and defective CD80 transendocytosis. Abatacept (CTLA-4-Ig) therapy effectively restored immune regulation and controlled disease activity. These findings expand the clinical and mechanistic spectrum of CTLA-4–related disorders, linking residual CTLA-4 function with the severity of immune dysregulation and emphasizing the therapeutic potential of targeted CTLA-4 modulation.

## Teaser

Catak et al. describe the first case of homozygous CTLA-4 deficiency, which drives severe early-onset autoimmunity through profound T and B cell dysregulation. They show that the variant impairs protein stability, yet targeted therapy with abatacept successfully restores immune balance, highlighting new clinical strategies for CTLA-4–related disorders.

## Introduction

Cytotoxic T lymphocyte–associated antigen 4 (CTLA-4; CD152) is a critical inhibitory receptor of the immunoglobulin superfamily, encoded by four exons, including a leader peptide sequence, ligand-binding domain, transmembrane region, and cytoplasmic tail, and composed of 223 amino acids (1, 2). CTLA-4 is transiently upregulated on activated effector T cells and constitutively expressed on regulatory T cells (Tregs), where it is indispensable for maintaining peripheral tolerance and preventing autoimmunity (3, 4, 5, 6). Functionally, CTLA-4 competes with the costimulatory receptor CD28 for binding to the shared ligands CD80 and CD86 on antigen-presenting cells (APCs) (7). While CD28 engagement delivers positive costimulatory signals that promote T cell proliferation and cytokine production, CTLA-4 engagement transduces inhibitory signals that restrain these responses (8, 9). Notably, CTLA-4 binds CD80 and CD86 with higher affinity than CD28 (7), and it can remove these ligands from the APC surface via transendocytosis, thereby attenuating CD28-mediated costimulation (10). Internalized CTLA-4 is subsequently recycled to the plasma membrane through clathrin-dependent endocytosis, ensuring sustained immune regulation (11). Consistent with these mechanistic insights, homozygous *Ctla4*-deficient mice exhibit severe lymphoproliferation and early lethality, whereas heterozygous animals are predominantly phenotypically normal (12, 13, 14). In humans, heterozygous *CTLA4* variants result in CTLA-4 insufficiency, an autosomal dominant disorder identified in 2014, which contrasts with the heterozygous asymptomatic condition observed in mice (15, 16).

Clinically affected individuals present with a spectrum of immune dysregulation, including lymphoproliferation, hypogammaglobulinemia, autoimmune cytopenias, enteropathy, and recurrent respiratory and gastrointestinal infections (15, 16, 17, 18, 19, 20). Immunological analyses demonstrate both numerical abnormalities and impaired suppressive capacity of Tregs, which is associated with expansion of memory T cells and autoreactive B cells (15, 16). Treatment with abatacept, a CTLA-4-Ig fusion protein, restores these cellular and clinical phenotypes and reverses dysregulated transcriptional and proteomic signatures, highlighting the potential of targeted costimulatory blockade in managing CTLA-4 insufficiency (17, 19, 20, 21).

While CTLA-4–related immune dysregulation is classically driven by heterozygous loss-of-function (LoF) alleles that result in haploinsufficiency, molecular disease mechanisms caused by missense mutations are far more diverse. Although some missense changes primarily destabilize protein folding, leading to classical LoF, others exert their effects via alternative mechanisms (22, 23). Because CTLA-4 functions as a homodimer and clusters at the immune synapse, specific missense alleles may impair ligand binding and/or dimerization, thereby exerting dominant-negative effects (16, 17). As observed in other disorders, distinct missense variants can produce hypomorphic alleles; these result in partial LoF that is clinically tolerated in heterozygous carriers but drives severe immune dysregulation when inherited in a homozygous biallelic manner (22, 23).

To our knowledge, although heterozygous *CTLA4* variants are well described, homozygous variants have not been reported in humans. Here, we present the first report providing a comprehensive mechanistic, cellular, and clinical characterization of homozygous CTLA-4 deficiency in humans. Additionally, we demonstrate that treatment with abatacept restores the immunological and clinical symptoms associated with this condition.

## Results

### A patient with early-onset immune dysregulation

**Figure 1.**
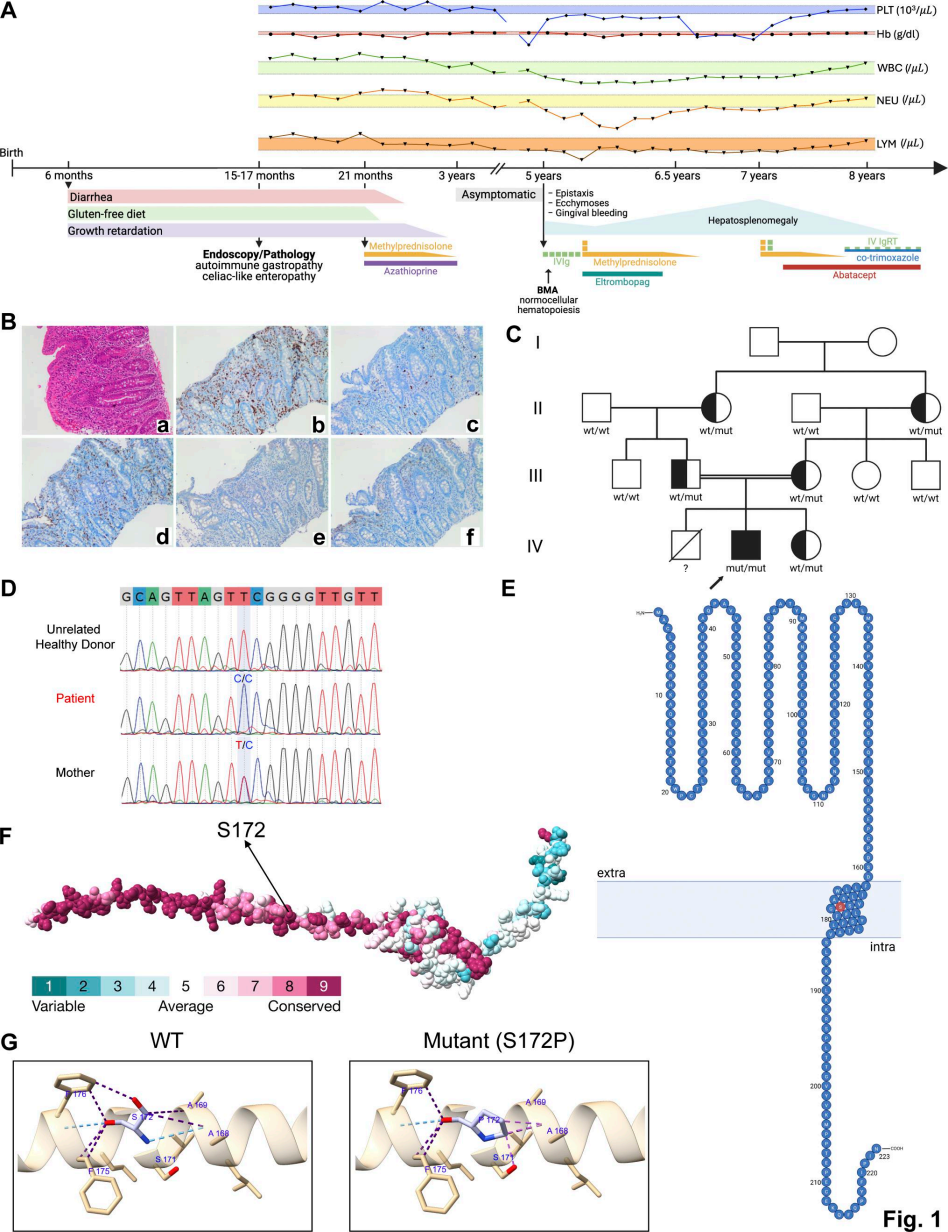
**Identification and structural characterization of the homozygous *CTLA4*** ^***S172P/S172P***^ **variant associated with immune dysregulation. (A)** Clinical timeline illustrating patient presentation, treatment interventions, and key laboratory findings. The colored shading represents the normal reference range for the respective parameter. **(B)** Histopathology of duodenal biopsy. (Panel a) Hematoxylin and eosin staining showing increased intraepithelial lymphocytes, mononuclear inflammatory cell infiltration in the lamina propria, irregular crypt architecture, and flattened/atrophic villi (20×), (Panel b) CD3 immunohistochemistry demonstrating dense T lymphocyte infiltration of the lamina propria and surface epithelium (20×), (Panel c) scattered and rare CD20-positive B lymphocytes in the lamina propria (20×), (Panel d) CD8 immunostaining highlighting cytotoxic T lymphocytes (20×), (Panel e) scattered and rare BCL6-positive B lymphocytes in the lamina propria (20×), (Panel f) PD-L1 immunostaining showing predominantly subsurface/near-luminal inflammatory cells, including lymphocytes and dendritic cells (20×). **(C)** Pedigree of the family showing inheritance pattern. **(D)** Sanger sequencing chromatograms of the proband, parents, and HC confirming the homozygous *CTLA4*: c.514T>C (p.S172P) variant. **(E)** Predicted structure of the CTLA-4 transmembrane domain obtained from the mCSM-membrane database, with the S172P mutant residue highlighted in red. **(F)** ConSurf-derived evolutionary conservation analysis mapped onto the AlphaFold-predicted CTLA-4 3D structure, indicating highly conserved residues in the vicinity of the S172P variant. **(G)** Structural comparison of WT and S172P mutant CTLA-4 generated in ChimeraX v1.10.1. Hydrogen bonds are colored light blue, hydrophobic contacts are purple, and steric clashes are depicted in pink, illustrating local conformational perturbations induced by the pathogenic variant. WT, wild-type; mut, mutant; PLT, platelet; Hb, hemoglabin; WBC, white blood cell; NEU, neutrophil; LYM, lymphocyte.

The patient is a 9-year-old boy, born full-term via cesarean section to consanguineous parents, weighing 4,100 g at birth, with an unremarkable neonatal course. Family history was notable for transient early-childhood diarrhea in his mother that resolved spontaneously by 3 years of age and the death of an older brother at 7 mo of age due to severe diarrhea of unknown etiology. His father and paternal uncle had congenital blindness, and genetic evaluation of the father revealed a homozygous retinal degeration 3 (*RD3*) variant (c.112C>T, p.Arg38*) consistent with Leber congenital amaurosis. The patient’s clinical history is shown in Fig. 1 A. His first symptom was nonbloody, nonmucoid diarrhea that began when he was introduced to complementary feeding at 6 mo of age. He experiences severe growth failure due to inadequate weight gain, linked to chronic diarrhea. Serological tests show positive anti-tissue transglutaminase IgA (185 U/ml) and anti-gliadin IgA levels (>200 U/ml), indicating celiac disease. As a result, he was started on a gluten-free diet. However, gastrointestinal symptoms and linear growth failure persisted and even worsened. An upper and lower gastrointestinal endoscopy was conducted at 15 mo of age, revealing persistent villous atrophy despite strict adherence to the gluten-free diet. The physical examination at 17 mo of age demonstrated significant growth failure, with length, weight, and head circumference all below the third percentile relative to standardized age-normal values.

An endoscopy was performed once more, and allergen testing was also performed because the symptoms continued. As a result of this evaluation, no specific food allergen was identified. Histopathological assessment of the duodenal biopsy demonstrated findings consistent with autoimmune gastropathy and celiac-like enteropathy associated with immune dysregulation. The biopsy showed significant intraepithelial lymphocytosis, mononuclear inflammatory cell infiltration in the lamina propria, irregular crypts, and flattened, atrophic villi. Immunohistochemical analysis revealed prominent CD3^+^ and CD8^+^ T cell infiltration in both the lamina propria and surface epithelium, while CD20^+^ and BCL6^+^ B cells were rare. PD-L1^+^ inflammatory cells, mainly lymphocytes and dendritic cells, were concentrated near the luminal surface (Fig. 1 B). Given the persistence of gastrointestinal symptoms despite strict adherence to a gluten-free diet, these findings were compatible with autoimmune, celiac-like enteropathy, supporting the initiation of immunosuppressive therapy. Therefore, at 21 mo of age, immunosuppressive therapy with methylprednisolone (1.5 mg/kg/day) and azathioprine (2 mg/kg/day) was initiated. Quantitative plasma PCR was performed at that time to determine the EBV DNA copy number, which was 2,495 IU/ml. Following the immunosuppressive therapy, there was a notable improvement in clinical symptoms. Due to the positive response, this treatment was continued for ∼1 year before being discontinued. The patient remained asymptomatic until 5 years of age, at which time he developed spontaneous ecchymoses, recurrent epistaxis, and gingival bleeding. Laboratory evaluation revealed thrombocytopenia and concurrent hepatosplenomegaly. He was diagnosed with immune thrombocytopenic purpura and treated with intravenous immunoglobulin (IVIG, 1 g/kg/dose). After six courses of IVIG without a meaningful hematologic response, two courses of pulse methylprednisolone (30 mg/kg/dose) were administered. Methylprednisolone was then continued at a maintenance dose of 2 mg/kg/day, and eltrombopag therapy (25 mg/day) was added, leading to a transient improvement in platelet counts over 2 mo. However, thrombocytopenia recurred while the patient was on corticosteroid therapy, and hepatosplenomegaly reappeared. Bone marrow examination demonstrated normocellular hematopoiesis with no evidence of malignancy or marrow failure. The thrombocytopenia was considered refractory to both IVIG and corticosteroids. At 6.5 years of age, the patient was reevaluated due to ongoing, treatment-refractory episodes of thrombocytopenia. It was noted that he had been receiving continuous methylprednisolone therapy for approximately 1 year, including intermittent pulse infusions. On physical examination, his height and weight were at the 25th percentile; however, he exhibited a prominent cushingoid appearance. The liver was palpable 5 cm below the right midclavicular line. Following discontinuation of eltrombopag and tapering of methylprednisolone, the patient experienced a severe relapse of thrombocytopenia at the age of 7 years. Two courses of pulse methylprednisolone therapy (30 mg/kg/day) and two courses of IVIG (1 g/kg) were administered, but no meaningful hematologic response was observed. While on maintenance methylprednisolone (2 mg/kg/day), the patient remained refractory to conventional immunosuppressive and supportive therapies. As part of the diagnostic workup, immune dysregulation was suspected. He exhibited low IgG levels; however, his IgA levels were elevated, and his IgM levels remained within the normal range before the IVIG replacement therapy. The detailed immunological, laboratory parameters, and vaccine response profiles are summarized in Table 1 and Fig. 1 A.

**Table 1.** Immunological evaluation of the *CTLA4*^*S172P/S172P*^ patient

Parameters	Before abatacept (Age: 7 years)	On abatacept (At 9 mo of therapy)	Reference values (5–10 years)
Leukocyte count (/μl)	**3,100 (↓)**	9,000	4,000–10,000
Absolute lymphocyte count (/μl)	**1,200 (↓)**	4,300	1,803–5,636
Absolute neutrophil count (/μl)	**1,100 (↓)**	4,000	2,000–7,000
Absolute eosinophil count (/μl)	110	210	20–500
Hemoglobin (g/dl)	11.2	14	11–16
Platelet (x10^3^/μl)	**6 (↓)**	315	150–450
IgG (mg/dl)	**677 (↓)**	On IgRT	764–2,134
IgA (mg/dl)	146	89	78–383
IgM (mg/dl)	74	**42 (↓)**	69–387
IgE (IU/ml)	3	7	<200
**Specific antibody titers**	​	​	​
Anti-HBs IgG (mIU/ml)	**<0.2 (−)**	On IgRT	<10 negative
			>= 10 positive
Anti-measles IgG (IU/L)	**0.82 (−)**	On IgRT	<0.9 negative
			0.9–1.1 borderline
			>1.1 positive
Anti-mumps IgG (U/ml)	1.51 (+)	On IgRT	<0.9 negative
			0.9–1.1 borderline
			>1.1 positive
Anti-varicella zoster IgG (S/CO)	**0.57 (−)**	On IgRT	<0.9 negative
			0.9–1.1 borderline
			>1.1 positive
**Lymphocyte subsets**	​	​	​
CD3^+^ T cells (%)	77	66	55–86
CD3^+^ count (/mm^3^)	**925 (↓)**	2,824	971–3,685
CD3^+^ CD4^+^ (%)	40	39	23–49
CD3^+^ CD4^+^ count (/mm^3^)	480	1,670	445–1,918
CD3^+^ CD8^+^ (%)	28	20	17–46
CD3^+^ CD8^+^ count (/mm^3^)	**336 (↓)**	856	379–2,084
CD19^+^ B cells (%)	16	**27 (↑)**	7–20
CD19^+^ count (/mm^3^)	192	**1,155 (↑)**	122–755
CD16 ^+^ 56^+^ NK cells (%)	**3 (↓)**	5	4–29
CD16 ^+^ 56^+^ NK cell count (/mm^3^)	**38 (↓)**	201	105–1,107
CD19^+^CD27^−^IgD^+^ B cells (%)	**89.1 (↑)**	**88 (↑)**	45–85
CD19^+^CD27^+^IgD^+^ B cells (%)	**2.8 (↓)**	**2.5 (↓)**	4–24
CD19^+^CD27^+^IgD^−^ B cells (%)	**1.1 (↓)**	**2.6 (↓)**	7–31
CD21^low^CD38^low^ activated B (%)	**30.3 (↑)**	9.8	2–14
CD3^+^ TCR^α/β^ cells (%)	77	85	68–96
CD3^+^ TCR^γ/δ^ cells (%)	12	11	5–30
CD4^+^CD45RA^+^CD31^+^ T cells (%)	**27.8 (↓)**	53.6	39–66
CD4^+^CD45RA^+^CCR7^+^ T cells (%)	**26.6 (↓)**	57.6	38–75
CD4^+^CD45RA^−^CCR7^+^ T cells (%)	27.1	35.1	14–49
CD4^+^CD45RA^−^CCR7^−^ T cells (%)	**38.4 (↑)**	6.1	2–16
CD4^+^CD45RA^+^CCR7^−^ T cells (%)	7.7	1.2	0–44
CD8^+^CD45RA^+^CCR7^+^ T cells (%)	28.1	**77.9 (↑)**	17–62
CD8^+^CD45RA^−^CCR7^+^ T cells (%)	5.3	**10.5 (↑)**	0–8
CD8^+^CD45RA^−^CCR7^−^ T cells (%)	**37 (↑)**	5.5	2–33
CD8^+^CD45RA^+^CCR7^−^ T cells (%)	29.6	6.1	14–61

IgRT, immunoglobulin replacement therapy; CD19^+^CD27^−^IgD^+^, naïve mature B cells; CD19^+^CD21^low^CD38^low^, autoreactive B cells; CD19^+^CD27^+^IgD^+^, non–class-switched memory B cells; CD19^+^CD27^+^IgD^−^, class-switched memory B cells; CD4^+^CD45RA^+^CCR7^+^, CD4^+^ naïve T cells; CD8^+^CD45RA^+^CCR7^+^, CD8^+^ naïve T cells; CD4^+^CD45RA^+^CD31^+^, recent thymic emigrants; CD4^+^CD45RA^−^CCR7^+^, central memory CD4^+^ T cells; CD4^+^CD45RA^−^CCR7^−^, effector memory CD4^+^ T cells; CD4^+^CD45RA^+^CCR7^−^, terminally differentiated effector memory CD4^+^ T cells; CD8^+^CD45RA^−^CCR7^+^, central memory CD8^+^ T cells; CD8^+^CD45RA^−^CCR7^−^, effector memory CD8^+^ T cells; CD8^+^CD45RA^+^CCR7^–^, terminally differentiated effector memory CD8^+^ T cells. Abnormal values are indicated boldly with upward (↑) or downward (↓) arrows in parentheses.

### Identification of a novel homozygous *CTLA4*^*S172P/S172P*^ variant

**Figure S1.**
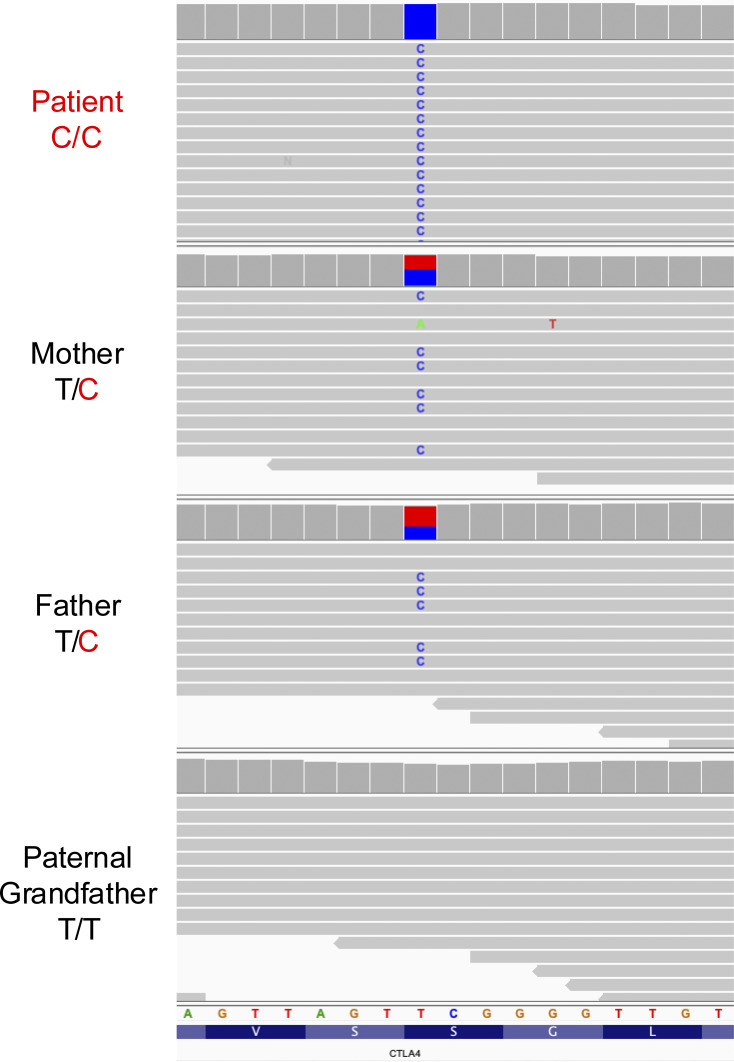
**WGS data viewed in IGV showing the CTLA4 mutation in the patient, parents, and grandfather.** The homozygous variant is present in the patient, whereas the parents carry the heterozygous variant. IGV, Integrative Genomics Viewer.

**Figure S2.**
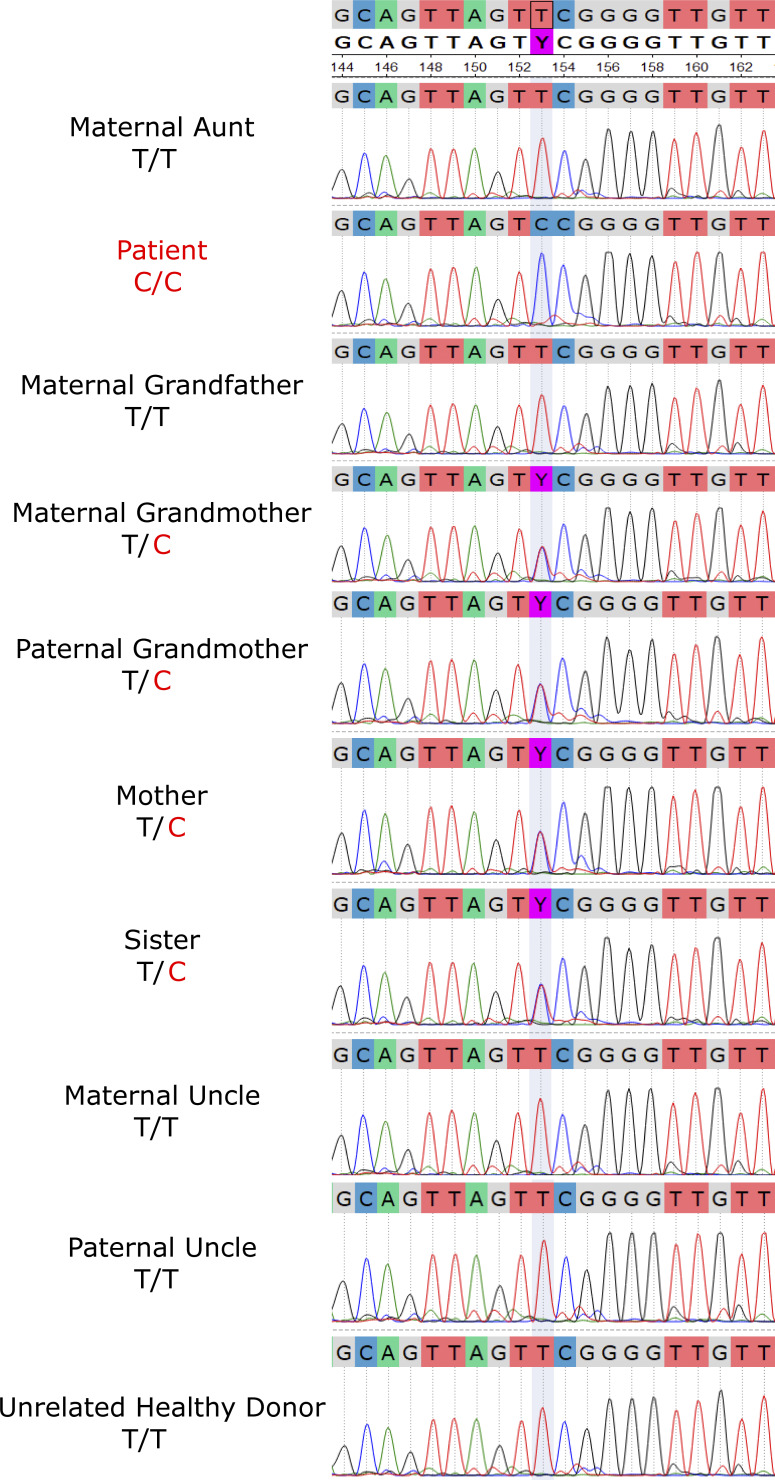
**Sanger sequencing chromatograms showing the CTLA4 variant in the extended family.** The homozygous variant is confirmed in the patient, demonstrating segregation from the heterozygous parents.

Whole-genome sequencing (WGS) performed on the patient and both parents (trio analysis) revealed a novel homozygous missense substitution in exon 3 of *CTLA4* (c.514T>C, p.S172P; NM_005214.5) (Fig. S1). Filtered candidate variants are listed in Table S1. Apart from *CTLA4*, none of the variants could explain the patient’s clinical manifestations, nor were they associated with immune system–related functions. This homozygous *CTLA4* variant, located on the predicted transmembrane domain, was confirmed by Sanger sequencing (Fig. 1, C–E). Variant segregation within the extended family is shown in Fig. S2. Clinical evaluation of the family revealed that all heterozygous carriers of the variant were completely asymptomatic, with no clinical signs of immune dysregulation. The ConSurf algorithm (using 144 homologous species sequences) and multiple sequence alignment by ClustalW demonstrated that the localization of this variant is a highly conserved region (Fig. 1 F). These results suggest a functional relevance of the variant region. Notably, the variant is absent from major population databases, including gnomAD, ExAC, and the 1000 Genomes Project, supporting its rarity and likely pathogenicity. The CADD score is 23.6, which is high and strongly supports a deleterious effect. The in silico pathogenicity predictors, including AlphaMissense (score 0.79, deleterious), MutationTaster (1.0, disease-causing), DANN (0.99, deleterious), and GenoCanyon (1.0, deleterious), consistently indicate that the variant is functionally damaging. Furthermore, the mutation Cutoff Scanning Matrix (mCSM)-membrane algorithm (24) predicts a destabilizing effect on protein stability (ΔΔG = −0.674 kcal/mol), reinforcing the pathogenic potential of the p.S172P substitution. Structurally, the wild-type (WT) Ser172 residue forms stabilizing hydrogen bonds with Ala168 and Phe176 and maintains additional contacts with Phe175, Phe176, Ala168, and Ala169 (Fig. 1 G). Substitution with proline disrupts the critical hydrogen bond with Ala168 and introduces steric clashes with Ala168 and Ser171, while partially preserving contacts with Phe175 and Phe176, collectively compromising local structural integrity (Fig. 1 G). Considering the functional findings consistent with CTLA-4 deficiency, treatment with intravenous abatacept was initiated at a dose of 10 mg/kg, administered as 3 loading doses at 2-wk intervals, followed by maintenance therapy every 4 wk. The patient exhibited a dramatic and sustained improvement in platelet counts following treatment with abatacept. During follow-up, immunoglobulin replacement therapy (0.5 g/kg/month) and cotrimoxazole prophylaxis were initiated to prevent infections. At the time of reporting, the patient was in the 20th mo of abatacept therapy and had not experienced any episodes of thrombocytopenia during this period.

### *CTLA4* ^*S172P/S172P*^ confers hypomorphic CTLA-4 expression and T cell hyperactivation

**Figure 2.**
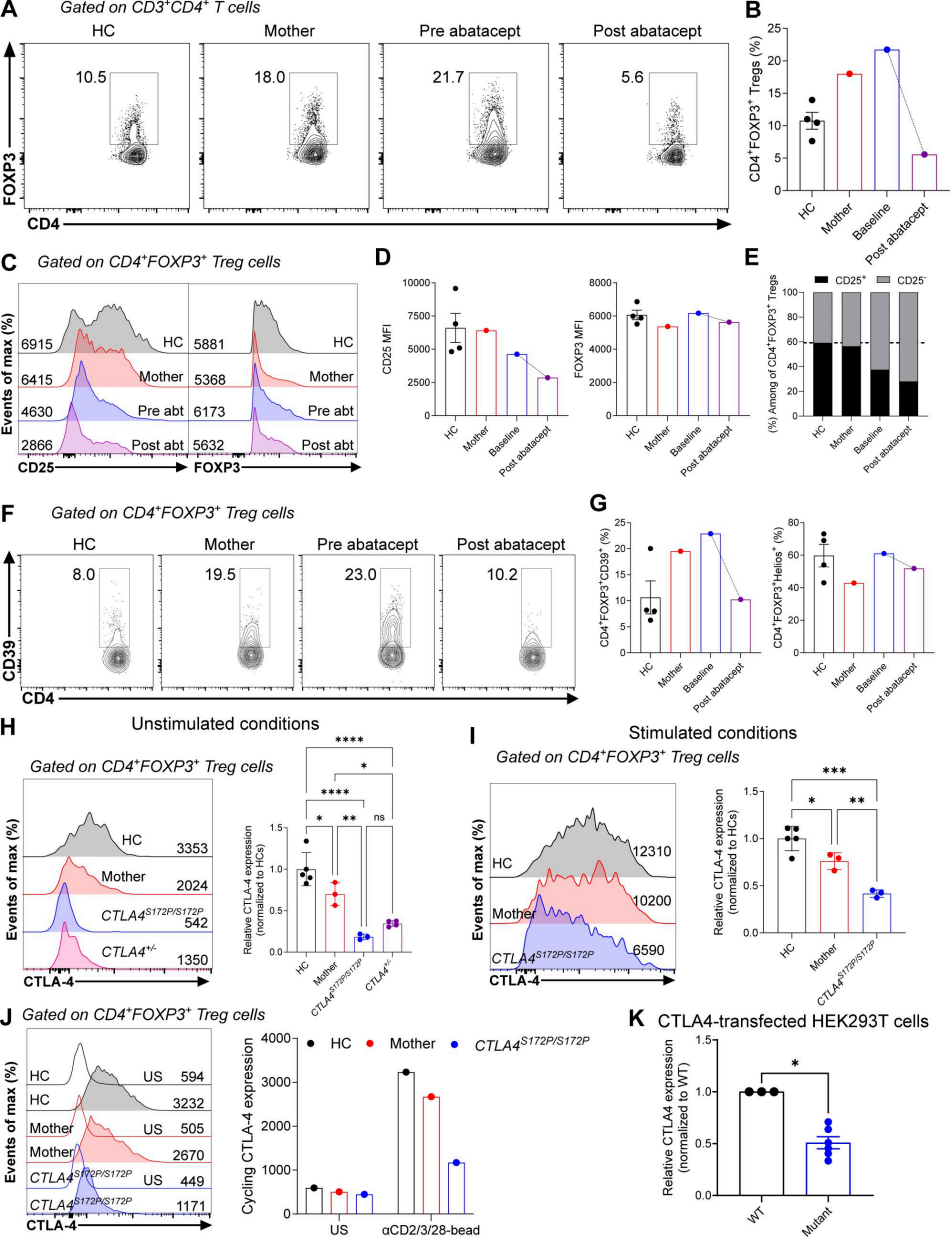
***CTLA4*** ^***S172P/S172P***^ **variant disrupts CTLA-4 expression in CD4** ^**+**^ **FOXP3** ^**+**^ **Tregs. (A)** Representative flow cytometry plot showing the frequency of CD4^+^FOXP3^+^ Tregs. **(B)** Bar graph depicting the percentages of CD4^+^FOXP3^+^ Tregs. **(C)** Representative histograms illustrating CD25 and FOXP3 expression levels within CD4^+^FOXP3^+^ Tregs. Indicated: gray, HC; blue, patient before abatacept (abt); purple, patient after abatacept (12 mo). **(D)** Bar graphs showing MFI of CD25 and FOXP3 in CD4^+^FOXP3^+^ Tregs. **(E)** Bar graph representing the proportion of CD25^+^ and CD25^−^ cells within the CD4^+^FOXP3^+^ Treg compartment. Indicated: black, CD25^+^; gray, CD25^−^. **(F)** Representative flow cytometry plot of CD39 expression within CD4^+^FOXP3^+^ Tregs. **(G)** Bar graphs showing the percentages of CD4^+^FOXP3^+^CD39^+^ and CD4^+^FOXP3^+^Helios^+^ Treg subsets. **(H)** Representative histograms of CTLA-4 expression in unstimulated CD4^+^FOXP3^+^ Tregs, with corresponding bar graphs of relative CTLA-4 expression normalized to HC. Black, HC; red, mother; blue, *CTLA4*^*S172P/S172P*^ patient; purple, CTLA-4–insufficient patients. **(I)** Representative histograms and normalized bar graphs showing CTLA-4 expression in stimulated CD4^+^FOXP3^+^ Tregs. Black, HC; red, mother; blue, *CTLA4*^*S172P/S172P*^ patient. **(J)** Representative histograms and relative bar graphs of recycling CTLA-4 expression in CD4^+^FOXP3^+^ Tregs, normalized to HC. Black, HC; red, mother; blue, *CTLA4*^*S172P/S172P*^ patient. Open histograms indicate US conditions, whereas filled histograms indicate stimulated conditions. **(K)** CTLA-4 levels in HEK293T cells overexpressing WT or the S172P mutant CTLA-4. Data are from three independent experiments, normalized to WT. Statistical significance was determined by the Wilcoxon signed-rank test. ns = nonsignificant, *P < 0.05, **P < 0.01, ***P < 0.001 and ****P < 0.0001. MFI, mean fluorescence intensity; US, unstimulated; WT, wild-type; HC, healthy contol.

CTLA-4 is a key molecule required for the suppressive capacity of Tregs; therefore, we evaluated the frequencies of Tregs and their canonical markers, including CD25, CTLA-4, Helios, and CD39. Interestingly, in contrast to previously reported CTLA4-haploinsufficient patients (21, 25), the *CTLA4*^*S172P/S172P*^ patient displayed an overall increase in CD4^+^FOXP3^+^ Treg frequencies, just as observed in homozygous *Ctla4-*deficient mice (Fig. 2, A and B) (26). However, CD25 expression was modestly reduced compared with healthy controls (HCs), while FOXP3 expression remained within the normal range (Fig. 2, C–E). Notably, the proportion of CD4^+^FOXP3^+^CD39^+^ Tregs was slightly elevated, whereas CD4^+^FOXP3^+^Helios^+^ Tregs were unaltered (Fig. 2, F and G). Following abatacept therapy, frequencies of CD4^+^FOXP3^+^ Tregs, CD4^+^FOXP3^+^CD39^+^ Tregs, and CD4^+^FOXP3^+^Helios^+^ Tregs declined (Fig. 2, A–G). Also, CD25 expression on CD4^+^FOXP3^+^ Tregs was reduced following abatacept therapy (Fig. 2, C–E). These changes result from the CD28 blockade by the CTLA-4-Ig (27).

We further assessed CTLA-4 expression on CD4^+^FOXP3^+^ Tregs to confirm the pathogenic impact of the homozygous S172P variant. The *CTLA4*^*S172P/S172P*^ patient had dramatically diminished, but residual, total CTLA-4 expression under unstimulated conditions compared with the HC and the heterozygous mother (Fig. 2 H). Interestingly, CTLA-4 expression in CTLA-4–haploinsufficient (*CTLA4*^*+/−*^) patients was not significantly different from that of the homozygous S172P patient, albeit S172P expression tended to be slightly lower.

Upon T cell activation with αCD2/CD3/CD28-coated beads in the presence of IL-2 for 5 days, inducible CTLA-4 upregulation remained markedly impaired in the homozygous patient (Fig. 2 I), whereas the heterozygous mother upregulated CTLA-4 to levels comparable to HCs, potentially explaining the absence of clinical disease in heterozygotes. Cycling CTLA-4 expression was similarly reduced in patient Tregs (Fig. 2 J), indicating defective intracellular trafficking and/or protein stability.

**Figure S3.**
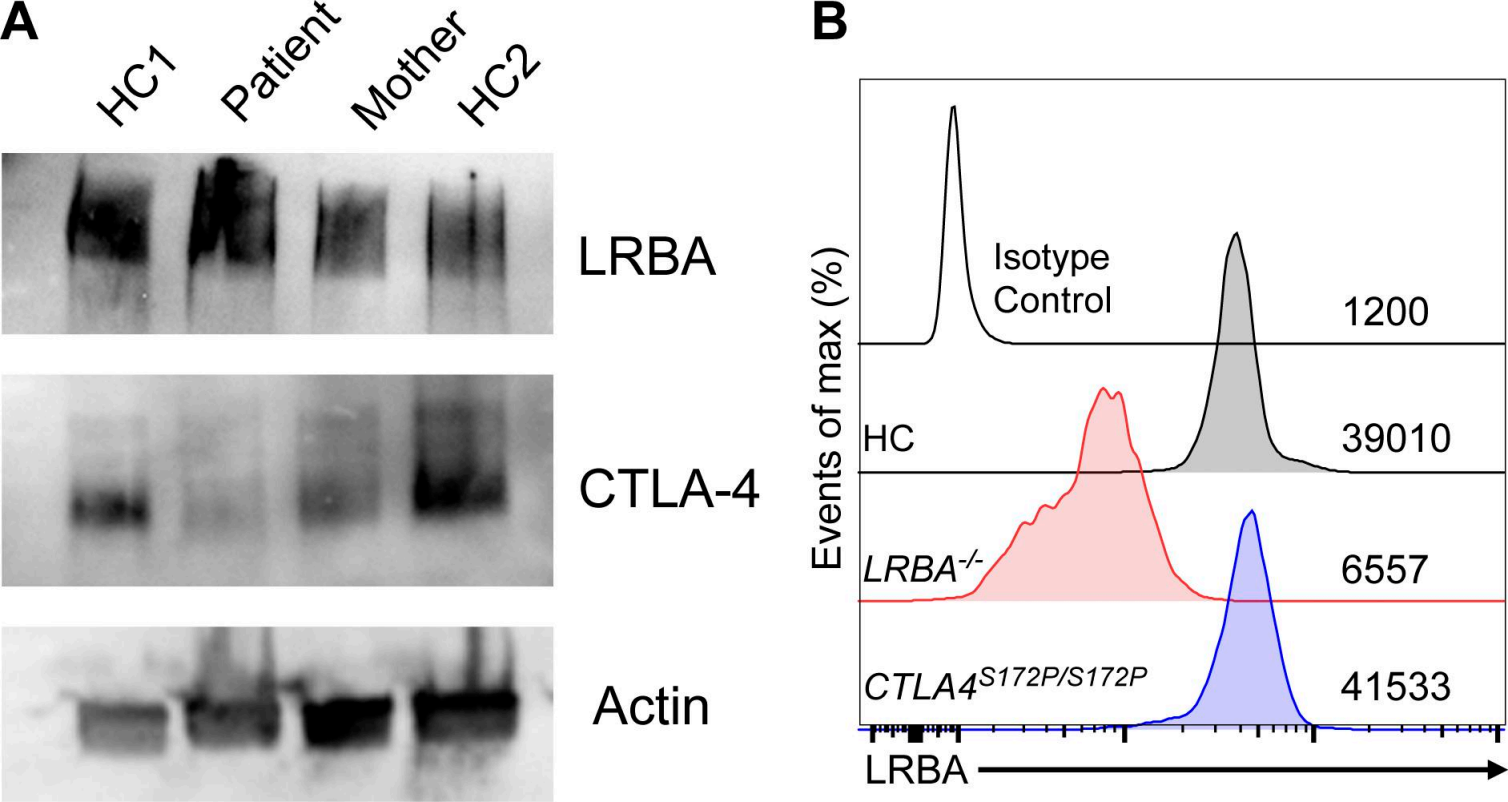
**Analysis of CTLA-4 and LRBA protein expression. (A)** Western blot analysis of CTLA-4 and LRBA protein levels in PBMCs activated with anti-CD3/CD28 for 4 days. Protein extracts from HCs, the heterozygous mother, and the patient were evaluated. **(B)** Representative flow cytometry histograms demonstrating intracellular LRBA expression in whole blood samples. Expression levels are compared among an HC, a patient with known LRBA deficiency, and the patient. HC, healthy control. Source data are available for this figure: SourceData FS3.

To rule out the possibility that the p.S172P substitution merely affected the antibody binding affinity that we used for staining, we also orthogonally validated the diminished CTLA-4 expression via western blot. Analysis of whole-cell lysates from peripheral blood mononuclear cells (PBMCs) stimulated with anti-CD3/CD28 for 4 days showed a marked reduction in total CTLA-4 protein in the patient compared with healthy donors (Fig. S3 A). Together, flow cytometry and immunoblot analyses establish that the S172P variant results in a severe quantitative defect in CTLA-4 expression while retaining residual protein, consistent with a hypomorphic allele rather than a null one.

Given that profound defects in CTLA-4 expression can arise secondarily from autosomal recessive LRBA mutations, particularly in consanguineous pedigrees, we sought to definitively exclude an underlying LRBA defect. Western blot analysis revealed that LRBA protein expression in the patient was intact and comparable to both the heterozygous mother and healthy donors (Fig. S3 A). To further corroborate this finding, we evaluated intracellular LRBA expression via flow cytometry. The patient exhibited normal LRBA levels, comparable to HCs and markedly higher than a confirmed LRBA-deficient patient (Fig. S3 B). Collectively, these results confirm that the diminished CTLA-4 expression is a primary consequence of the homozygous *CTLA4* variant rather than a secondary defect resulting from LRBA loss.

To further assess the impact of the S172P variant on CTLA4 protein expression, we cloned the variant and overexpressed both WT and the S172P mutant CTLA4 in HEK293T cells. Our analysis revealed that the S172P mutant is expressed but at reduced levels compared with WT CTLA4 (Fig. 2 K). These findings also confirm that the S172P variant is hypomorphic, retaining partial protein expression.

**Figure 3.**
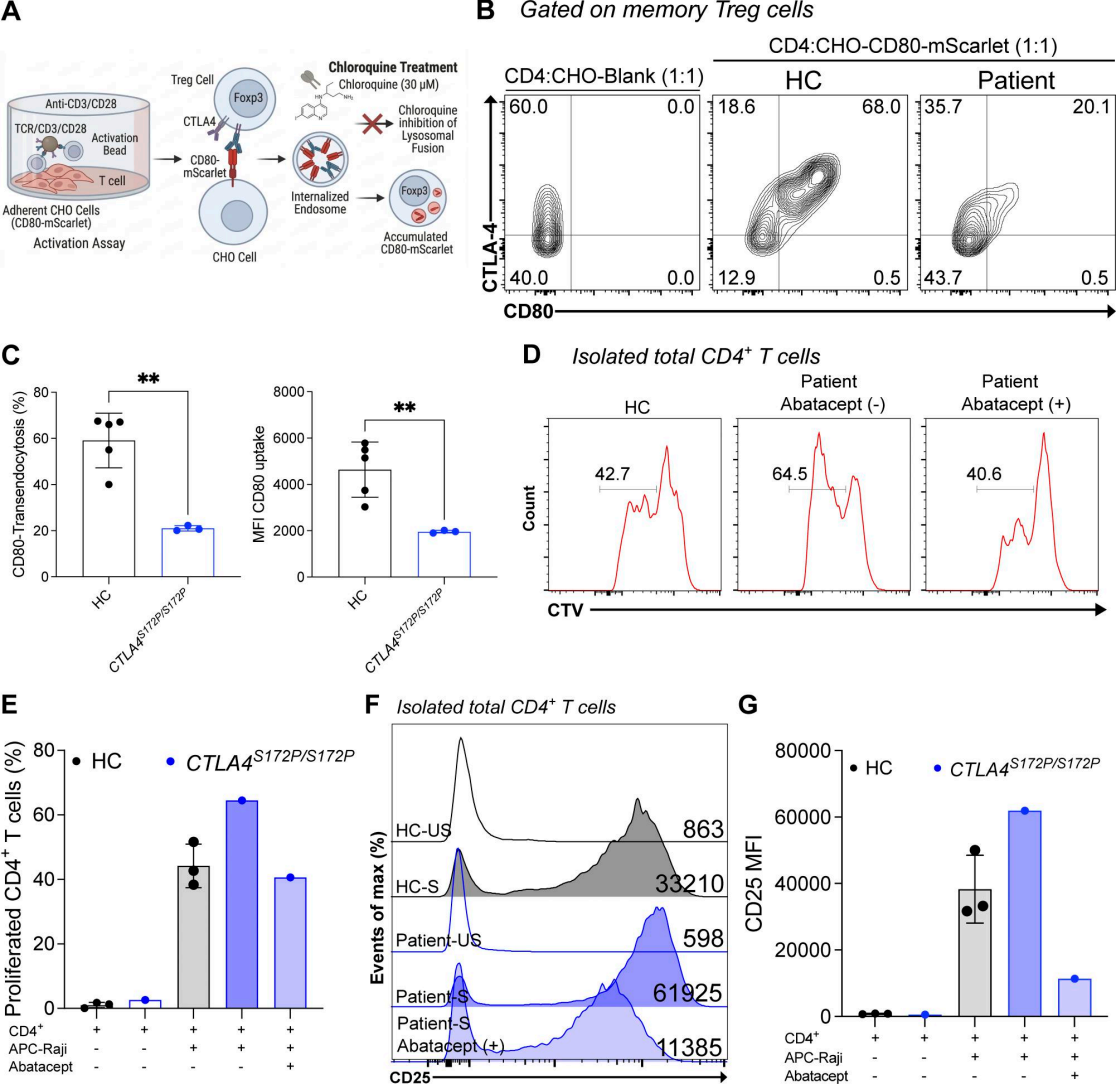
***CTLA4*** ^***S172P/S172P***^ **variant severely impairs CD80 transendocytosis and drives CD4** ^**+**^ **T cell hyperproliferation. (A)** Schematic representation depicting the principle of the in vitro CD80 transendocytosis assay. **(B)** Representative flow cytometry plots showing CD80 internalization in cells from a HC versus the patient. **(C)** Quantitative bar graphs showing the percentages of CD80 transendocytosis and the MFI of CD80 uptake. **(D)** Representative histograms showing proliferation of CD4^+^ T cells isolated from HC and patient after 4-day coculture with mitomycin C–treated APC-Raji cells. **(E)** Bar graph depicting the percentages of proliferating CD4^+^ T cells. **(F)** Representative histograms of CD25 expression on CD4^+^ T cells after stimulation. **(G)** Bar graphs showing the mean MFI of CD25 expression on CD4^+^ T cells. In panel C, an unpaired *t* test was used to analyze significance. **P < 0.01. MFI, mean fluorescence intensity; APC, antigen-presenting cells; HC, healthy control, US, unstimulated; S, stimulated; CHO, chinese hamster ovary.

To evaluate the functional consequences of the variant on the extrinsic regulatory capacity of CTLA-4, we assessed its ability to capture and internalize CD80 via transendocytosis. The experimental workflow and principle of this assay are depicted in Fig. 3 A. Briefly, isolated CD4^+^ T cells were cocultured for 16 h with a Chinese hamster ovary (CHO) cell line stably expressing a CD80-mScarlet fusion protein and continuously stimulated with anti-CD2/CD3/CD28-coated beads. Following co-incubation, the intracellular accumulation of CD80-mScarlet was quantified specifically within the memory Treg compartment using flow cytometry. Consistent with diminished protein expression, patient memory Tregs exhibited a profound impairment in transendocytosis compared with the robust CD80 acquisition observed in HCs, demonstrating a dramatic and statistically significant reduction in internalized CD80-mScarlet levels (Fig. 3, B and C). These findings functionally confirm that the homozygous variant severely abrogates the suppressive capability of Tregs.

To assess T cell activation driven by CTLA-4 deficiency, CD4^+^ T cells were isolated using a magnetic bead separation method and subsequently cocultured with mitomycin C–treated APC/Raji B cells (J159A; Promega). In this setting, the patient’s CD4^+^ T cells exhibited increased proliferation and CD25 expression compared with those of HCs (Fig. 3, D–G). The addition of abatacept to the coculture led to a pronounced reduction of both proliferative responses and CD25 upregulation, indicating effective inhibition of excessive costimulatory signaling (Fig. 3, D–G). These results suggest that the loss of CTLA-4 expression underlies the hyperproliferative phenotype observed in the patient.

### Enhanced CTLA-4 degradation caused by the *CTLA4*^*S172P/S172P*^ variant was reversed upon lysosomal degradation inhibition

We investigated the mechanism underlying CTLA-4 reduction by assessing CTLA-4 protein stability. To determine the protein’s half-life, a cycloheximide (CHX) chase assay was performed. PBMCs from the *CTLA4*^*S172P/S172P*^ patient and HCs were expanded with αCD2/CD3/CD28-coated beads in the presence of IL-2 for 5 days. Following activation, cells were treated with CHX (30 µg/ml) and subsequently fixed, permeabilized, and stained for CD3, CD4, FOXP3, and CTLA-4 at 0, 2, 4, and 6 h.

**Figure 4.**
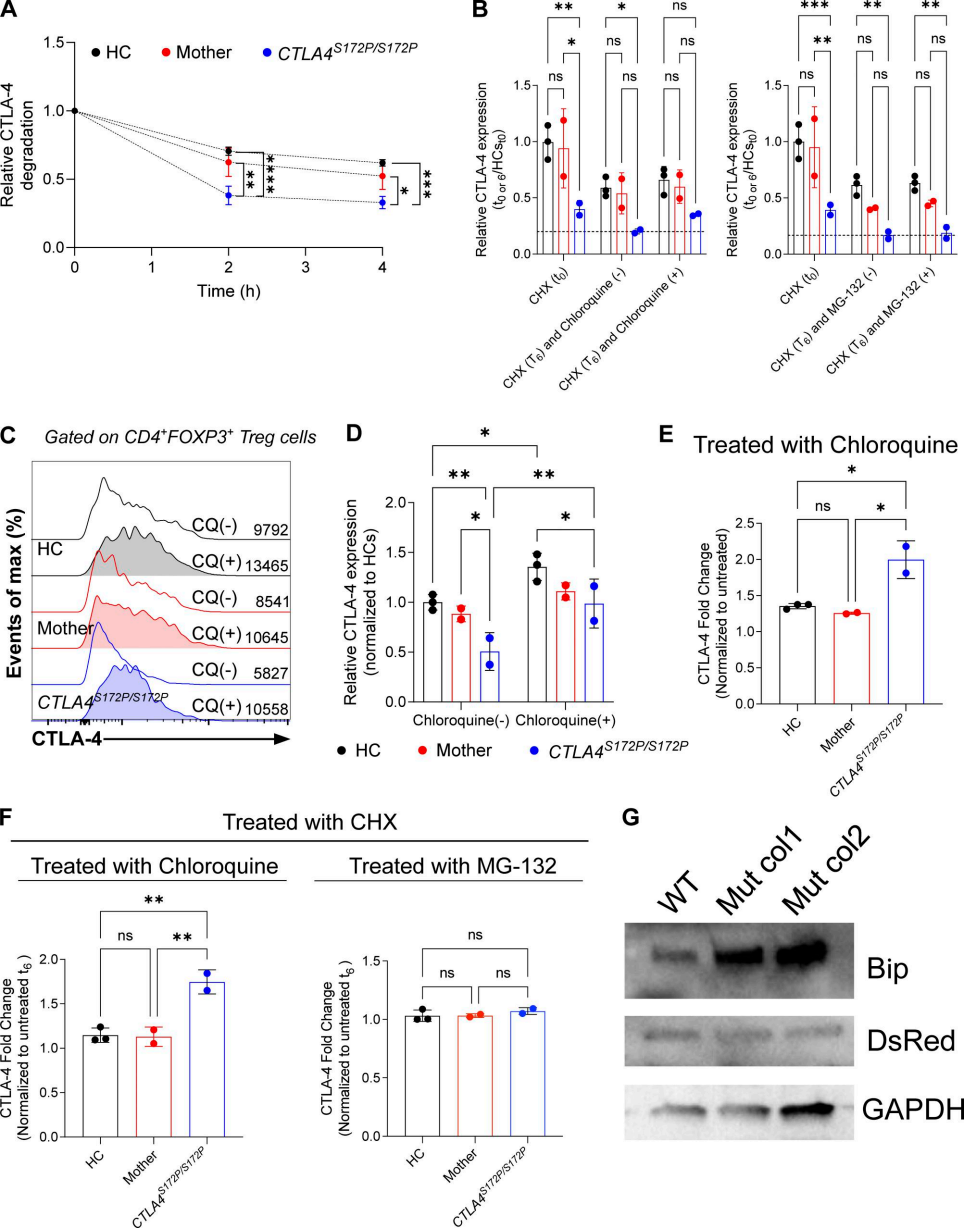
***CTLA4*** ^***S172P/S172P***^ **variant accelerates the lysosomal degradation of CTLA-4. (A)** PBMCs were treated with 30 μg/ml CHX for up to 4 h at 37°C and stained for total CTLA-4. The graph shows total CTLA-4 expression relative to 0 h, indicating relative CTLA-4 degradation. **(B)** Relative CTLA-4 expression after 6 h of CHX treatment with or without chloroquine and MG-132, normalized to HCs. **(C)** Representative histograms showing CTLA-4 expression in PBMCs treated with chloroquine for 6 h compared with untreated conditions. **(D)** Bar graphs illustrating CTLA-4 expression after 6 h of chloroquine treatment, normalized to HC. **(E)** Fold change in CTLA-4 expression after 6 h of chloroquine treatment, normalized to untreated conditions. **(F)** Bar graphs showing CTLA-4 fold change following 6 h of CHX treatment with or without chloroquine or MG-132, normalized to untreated conditions. **(G)** Immunoblot analysis of the ER stress marker BiP in cells transfected with WT CTLA-4 and two independent S172P mutant clones. GAPDH was used as a loading control. Statistical significance was assessed using two-way ANOVA with Tukey’s post hoc test for Panels (A, B, and D). In panel F, one-way ANOVA with Tukey’s post hoc test was used to analyze significance. ns = nonsignificant, *P < 0.05, **P < 0.01, ***P < 0.001, **** P < 0.0001. ER, endoplasmic reticulum; HC, healthy control; WT, wild-type; Mut, mutant; col, colony; CHX, cycloheximide; CQ, chloroquine. Source data are available for this figure: SourceData F4.

CTLA-4 degradation kinetics revealed a significantly accelerated loss of CTLA-4 in the patient’s Tregs compared with both HC and the heterozygous mother (Fig. 4 A), indicating reduced protein stability associated with the S172P variant.

To delineate the degradation pathway, activated T cells were treated with the lysosomal inhibitor chloroquine (50 µM) or the proteasomal inhibitor MG-132 (10 µM) for 6 h, in the presence or absence of CHX. MG-132 had no significant effect on CTLA-4 expression, whereas chloroquine treatment resulted in a marked increase in CTLA-4 levels in the patient’s Tregs (Fig. 4, B–F), indicating preferential lysosomal degradation.

Notably, while chloroquine substantially restored CTLA-4 expression in the patient’s cells, it had minimal impact on cells from the heterozygous mother and HC. This suggests that in the heterozygous state, CTLA-4 is not subject to enhanced lysosomal degradation, consistent with preserved protein stability.

We next sought to define the upstream cellular pathways that route mutant CTLA-4 to the lysosome. Hypothesizing that the p.S172P variant induces structural misfolding, we evaluated markers of endoplasmic reticulum stress. Indeed, ectopic expression of the S172P mutant induced higher levels of the chaperone protein BiP compared with WT CTLA-4. These findings, robustly confirmed across three separate transfections utilizing two independently derived mutant clones, suggest that the misfolded mutant protein activates the unfolded protein response, leading to its subsequent lysosomal degradation (Fig. 4 G).

### Abatacept treatment corrects T follicular helper cells, and memory T and B cell dysregulation driven by the *CTLA4*^*S172P/S172P*^ variant

**Figure 5.**
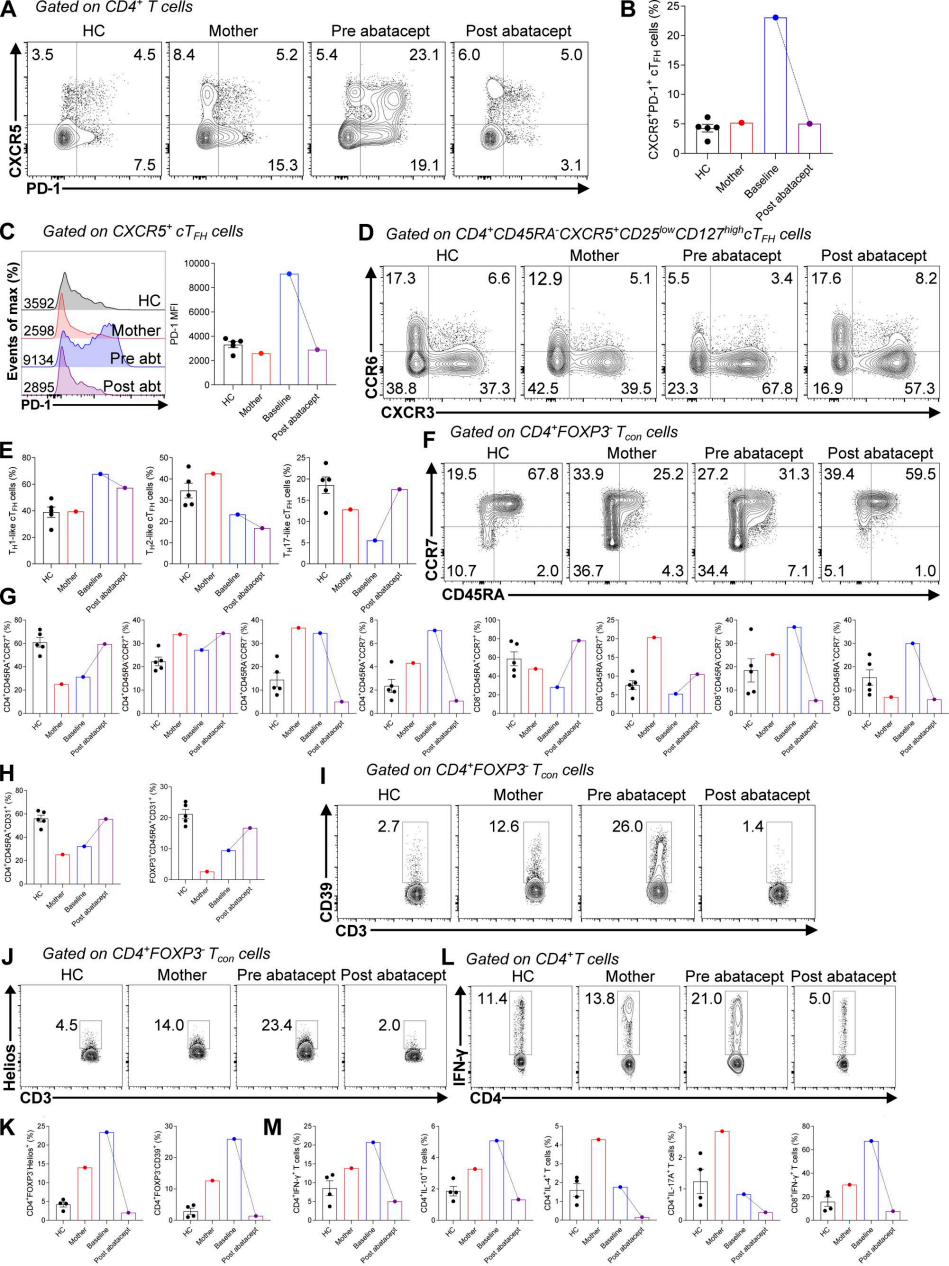
**Abatacept restores T cell homeostasis disrupted by the *CTLA4*** ^***S172P/S172P***^ **variant. (A)** Representative flow cytometry plots of CXCR5^+^PD-1^+^ cT_FH_ cells comparing HCs, mother, and patient before and after abatacept treatment (12 mo). **(B)** Percentages of CXCR5^+^PD-1^+^ cT_FH_ cell frequencies across HC, mother, and patient before and after abatacept (12 mo). **(C)** Representative histograms showing PD-1 MFI on CXCR5^+^ cT_FH_ cells, with bar graphs summarizing MFI values in HC, mother, and patient before and after abatacept (abt) (12 mo). **(D)** Representative plots depicting cT_FH_ subset distribution based on T_H_1 (CXCR3^+^CCR6^−^), T_H_2 (CXCR3^−^CCR6^−^), and T_H_17 (CXCR3^−^CCR6^+^) phenotypes in all donors. **(E)** Bar graphs showing the percentages of T_H_1, T_H_2, and T_H_17 cT_FH_ subsets in HC, mother, and patient before and after abatacept therapy. **(F)** Representative plots illustrating CD4^+^FOXP3^−^ T_con_ cell differentiation into naïve (CD45RA^+^CCR7^+^), central memory (CD45RA^−^CCR7^+^), effector memory (CD45RA^−^CCR7^−^), and EMRA (CD45RA^+^CCR7^−^) compartments across the donors. **(G)** Bar graphs depicting the percentages of naïve, central memory, effector memory, and EMRA CD4^+^FOXP3^−^ T_con_ and CD8^+^ T cells in HC, mother, and patient before and after abatacept. **(H)** Frequencies of recent thymic emigrants (CD3^+^CD45RA^+^CD31^+^) within CD4^+^FOXP3^−^ T_con_ and CD4^+^FOXP3^+^ Tregs across all donors. **(I)** Representative plots of CD39 expression within CD4^+^FOXP3^−^ T_con_ cells in HC, mother, and patient before and after treatment. **(J)** Representative plots showing Helios expression in CD4^+^FOXP3^−^ T_con_ cells across donors and treatment conditions. **(K)** Bar graphs showing the percentages of CD39^+^ and Helios^+^ T_con_ cells in HC, mother, and patient before and after abatacept. **(L)** Representative flow cytometry plots of IFN-γ^+^ CD4^+^ T cells across HC, mother, and patient before and after abatacept. **(M)** Bar graphs showing the proportions of cytokine-producing T cells (CD4^+^IFN-γ^+^, CD4^+^IL-10^+^, CD4^+^IL-4^+^, CD4^+^IL-17A^+^, and CD8^+^IFN-γ^+^) in HC, mother, and patient before and after abatacept. HC, healthy control, MFI, mean fluorescence intensity.

PBMCs from a *CTLA4*^*S172P/S172P*^ patient were analyzed to assess immune cell phenotypes at baseline and 12 mo following abatacept therapy. We have previously reported that *CTLA4*^*+/−*^ patients exhibit an increased frequency of circulating T follicular helper (cT_FH_) cells with elevated PD-1 expression, indicative of aberrant activation (21, 25). Consistent with these findings, the *CTLA4*^*S172P/S172P*^ patient demonstrated markedly expanded cT_FH_ cells with high PD-1 expression, which progressively normalized under abatacept treatment (Fig. 5, A–C). Phenotypic profiling further revealed that these dysregulated cT_FH_ cells were skewed toward a T helper (T_H_) 1–like (CXCR3^+^CCR6^−^) phenotype, while T_H_2-like (CXCR3^−^CCR6^−^) and T_H_17-like (CXCR3^−^CCR6^+^) subsets were diminished (Fig. 5, D and E). Notably, abatacept treatment gradually corrected this imbalance, with the most pronounced recovery observed in the T_H_17-like compartment (Fig. 5, D and E).

**Figure S4.**
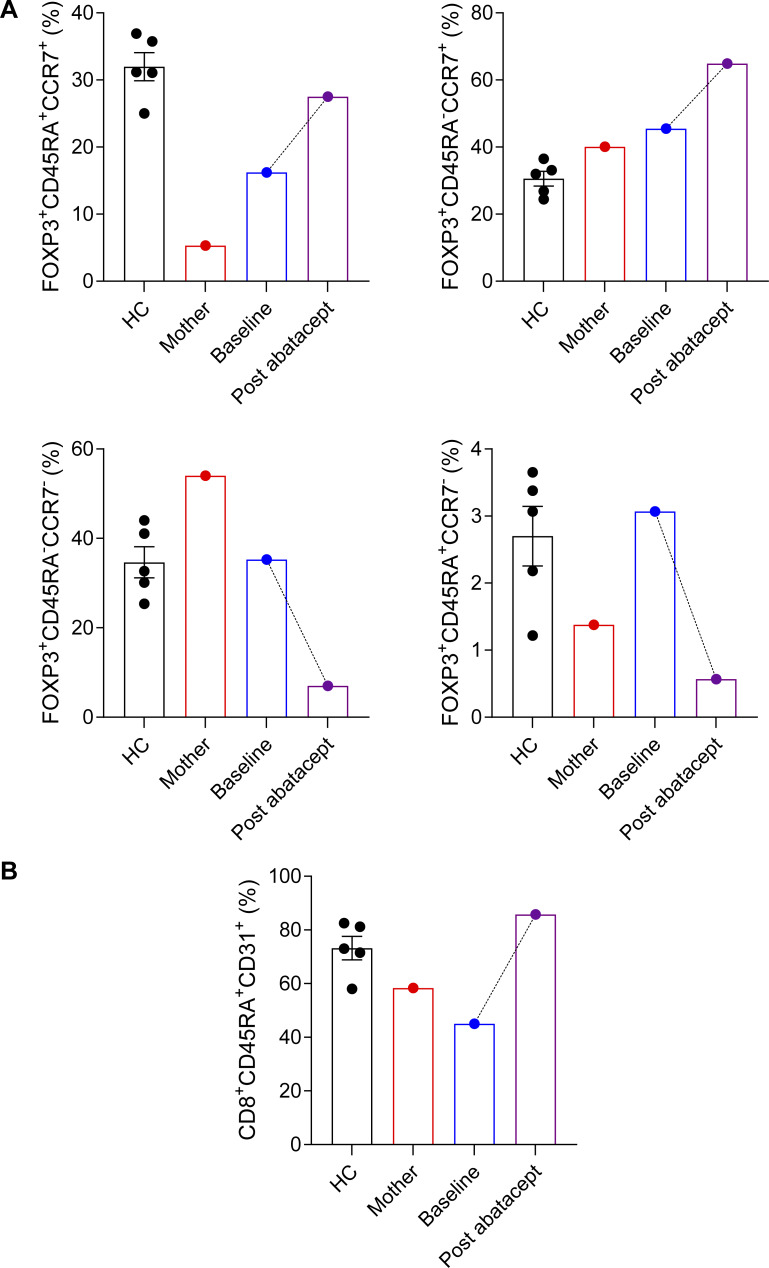
**Changes in T cell population with abatacept treatment. (A)** Frequencies of Treg subsets, including naïve (CD4^+^FOXP3^+^CD45RA^+^CCR7^+^), central memory (CD4^+^FOXP3^+^CD45RA^−^CCR7^+^), effector memory (CD4^+^FOXP3^+^CD45RA^−^CCR7^−^), and effector memory RA (CD4^+^FOXP3^+^CD45RA^+^CCR7^−^) populations, are shown for HC, mother, and the patient before and after treatment. **(B)** Proportion of CD8^+^CD45RA^+^CD31^+^ T cells in HC, mother, and the patient before and after treatment. HC, healthy control.

At baseline, the *CTLA4*^*S172P/S172P*^ patient exhibited elevated frequencies of effector memory (EM; CD45RA^−^CCR7^−^) and effector memory RA (EMRA; CD45RA^+^CCR7^−^) T cells, along with a marked reduction in the naïve T cell pool, across conventional CD4^+^ T helper (T_con_), Treg, and CD8^+^ T cell subsets (Fig. 5, F and G; and Fig. S4 A). These cell types also displayed a decreased proportion of CD31^+^ recent thymic emigrants, suggesting impaired thymic output or homeostatic imbalance (Fig. 5 H and Fig. S4 B). Significantly, these aberrant phenotypes were progressively normalized following abatacept therapy (Fig. 5, F–H; and Fig. S4, A and B). Additionally, we examined the expression of CD39 and Helios on T_con_ cells. We found that the *CTLA4*^*S172P/S172P*^ patient had a markedly elevated frequency of CD39 and Helios, and this phenotype was profoundly normalized following treatment (Fig. 5, I–K).

Additionally, we evaluated intracellular cytokine production in the patient’s CD4^+^ and CD8^+^ T cells. Our previous study demonstrated that *CTLA4*^*+/−*^ patients exhibit elevated production of IFN-γ and IL-10 by CD4^+^ T cells, both of which were markedly reduced following abatacept therapy (21). Consistently, in the *CTLA4*^*S172P/S172P*^ patient, a similar cytokine pattern was observed, with increased IFN-γ and IL-10 production by CD4^+^ T cells that declined upon treatment (Fig. 5, L and M). Moreover, IL-4 and IL-17A production by CD4^+^ T cells was initially within the normal range but was likewise reduced after therapy, reflecting a comparable immunomodulatory response to CTLA4-Ig–mediated costimulatory blockade (Fig. 5 M). Furthermore, IFN-γ production of CD8^+^ T cells was elevated at baseline; however, abatacept therapy restored this aberrant cytokine response (Fig. 5 M). These results indicate that abatacept effectively corrects dysregulated cytokine responses in the patient.

**Figure S5.**
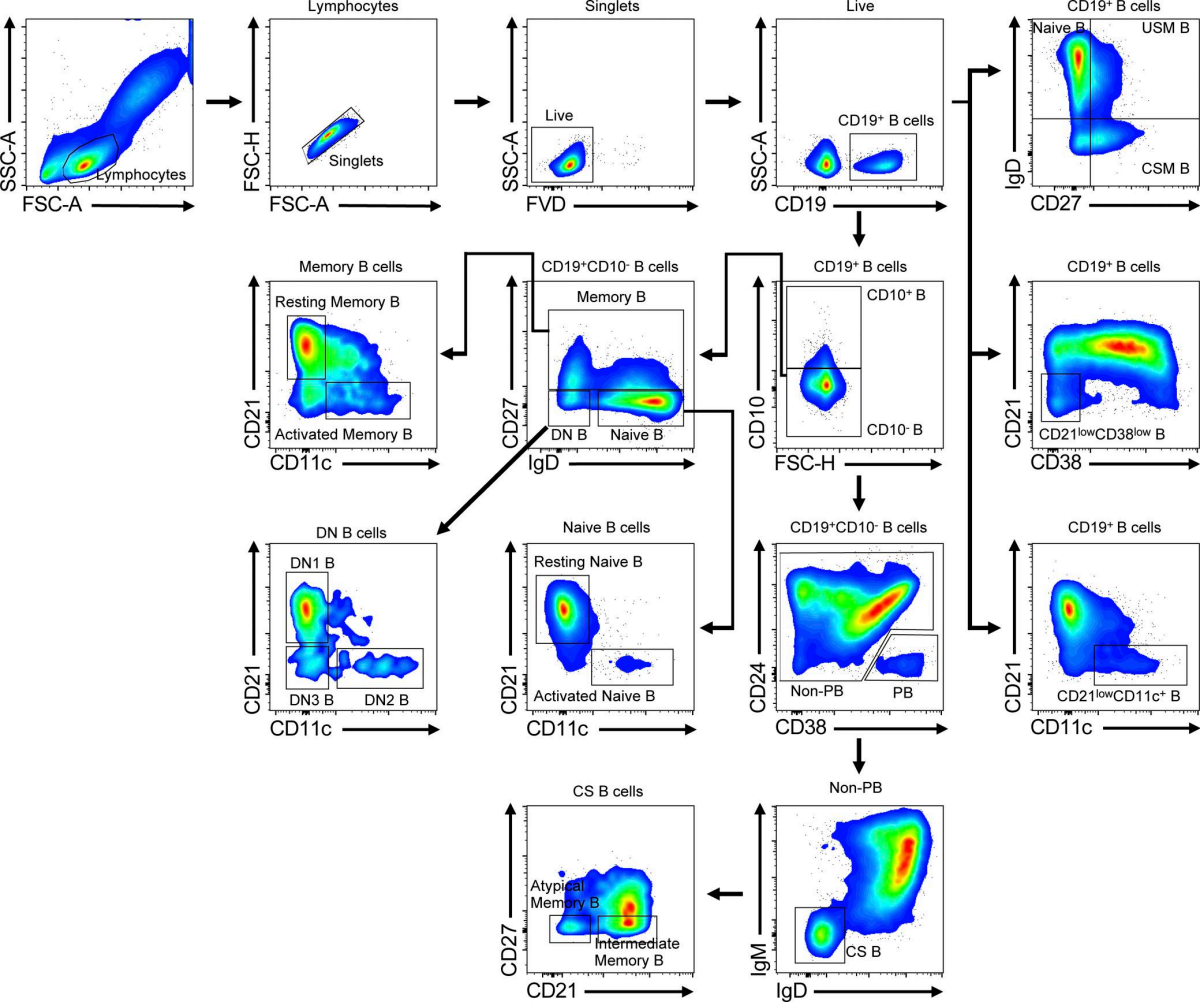
**Gating strategy for B cell immunophenotyping by flow cytometry. **USM, unswitched memory; CSM, class-switched memory; CS, class-switched; PB, plasmablast; DN, double-negative; FVD, fixable viability dye.

**Figure 6.**
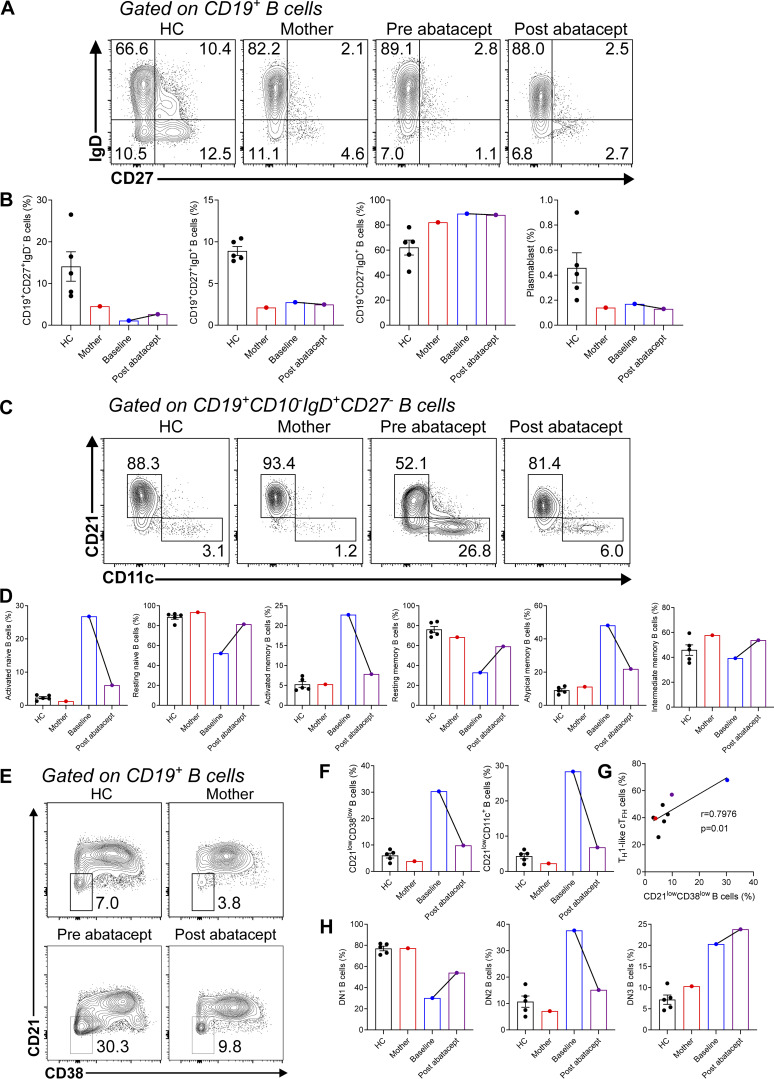
**Abatacept partially normalizes B cell phenotype and reduces activated cell subsets in the *CTLA4*** ^***S172P/S172P***^ **patient. (A)** Representative plots illustrate naïve B cells (IgD^+^CD27^−^), class-switched memory B cells (IgD^−^CD27^+^), and unswitched memory B cells (IgD^+^CD27^+^) across HCs, mothers, and patients before and after abatacept treatment (12 mo). **(B)** Bar charts display the percentages of naïve, class-switched memory, unswitched memory B cells, and plasmablasts in HC, mothers, and patients before and after treatment. **(C)** Representative plots show activated naïve B cells (CD19^+^CD10^−^IgD^+^CD27^−^CD21^−^CD11c^+^) and resting naïve B cells (CD19^+^CD10^−^IgD^+^CD27^−^CD21^+^CD11c^−^). **(D)** Bar graphs depict the proportions of activated naïve, resting naïve, activated memory, resting memory, atypical memory, and intermediate memory B cells among donors. **(E)** Representative plots illustrate CD19^+^CD21^low^CD38^low^ B cells. **(F)** Bar charts show percentages of CD21^low^CD38^low^ B cells and CD21^low^CD11c^+^ B cells in HC, mothers, and patients before and after abatacept. **(G)** Correlation analysis between T_H_1-like cT_FH_ cells and CD21^low^CD38^low^ B cell frequencies was performed using Spearman’s correlation. The color codes are blue for patient before abatacept, purple for patient after abatacept, red for mother, and black for HC. **(H)** Distribution of DN1, DN2, and DN3 B cell subsets across all donors is analyzed before and after abatacept treatment.

Furthermore, we performed detailed immunophenotyping of B cells, and the gating strategy is presented in Fig. S5. *CTLA4*^*S172P/S172P*^ patient revealed an expansion of naïve B cell (CD19^+^CD27^−^IgD^+^) frequency accompanied by a reduction in class-switched (CD19^+^CD27^+^IgD^−^) and unswitched memory (CD19^+^CD27^+^IgD^+^) B cell frequency with a slight decrease in plasmablasts (CD19^+^CD10^−^CD24^low^CD38^high^) (Fig. 6, A and B). Abatacept therapy had no significant effect on the distribution of these major B cell subtypes (Fig. 6, A and B). Detailed phenotypic analysis of naïve B cells showed that activated naïve B cells (CD19^+^CD10^−^IgD^+^CD27^−^CD21^−^CD11c^+^) were increased, while resting naïve B cells (CD19^+^CD10^−^IgD^+^CD27^−^CD21^+^CD11c^−^) were decreased (Fig. 6, C and D). Similarly, within the memory B cell compartment, atypical (CD19^+^CD10^−^CD38^low^IgM^-^IgD^-^CD21^−^CD27^−^) and activated memory B (CD19^+^CD10^−^CD27^+^CD21^−^CD11c^+^) cells were notably elevated, while resting memory B cells (CD19^+^CD10^−^CD27^+^CD21^+^CD11c^−^) were diminished, and intermediate memory B cells (CD19^+^CD10^−^CD38^low^IgM^−^IgD^−^CD21^+^CD27^−^) remained comparable to HCs (Fig. 6 D). Moreover, CD21^low^CD38^low^ B and CD21^low^CD11c^+^ cells were elevated before the abatacept therapy (Fig. 6, E and F). CD21^low^CD38^low^ B cells correlate positively with T_H_1-like cT_FH_ cells (Fig. 6 G). However, these dysregulated B cell phenotypes were restored by the abatacept therapy (Fig. 6, A–F). Furthermore, abatacept treatment corrected the reduction in double-negative (DN) 1 B cells (CD19^+^CD10^-^IgD^−^CD27^−^CD21^+^CD11c^−^) and normalized the expansion of DN2 B cells (CD19^+^CD10^−^IgD^−^CD27^−^CD21^−^CD11c^+^), whereas DN3 B cells (CD19^+^CD10^−^IgD^−^CD27^−^CD21^−^CD11c^−^) persisted at elevated frequencies despite therapy (Fig. 6 H).

## Discussion

CTLA-4 is a critical inhibitory receptor that regulates T cell activation and maintains immune homeostasis. Heterozygous LoF mutations in *CTLA4* cause CTLA-4 insufficiency, leading to variably penetrant immune dysregulation; however, biallelic CTLA-4 deficiency has not previously been documented in humans. Human CTLA-4 insufficiency was first described in 2014, and since then, several heterozygous variants have been reported. In 2026, approximately a decade later, we report one of the first known cases of a homozygous *CTLA4* variant. Notably, the p.S172P substitution is hypomorphic rather than null, as it allows partial CTLA-4 expression. These findings also imply that complete homozygous loss of CTLA4 function is likely incompatible with life in humans. In our patient, the homozygous S172P variant resulted in immune dysregulation characterized by hyperactivation of T and B cells and dysregulated cytokine production. Remarkably, abatacept therapy effectively restored immunological balance, emphasizing the indispensable role of CTLA-4 signaling in maintaining human immune tolerance.

Our patient exhibits a phenotype that is markedly distinct from that observed in homozygous *Ctla4*-deficient mouse models. While Ctla4 knockout mice develop fatal lymphoproliferation characterized by extensive T cell infiltration and multi-organ tissue destruction within weeks of birth (12, 13, 14), our patient presented with a comparatively attenuated, nonlethal phenotype. This discrepancy likely reflects minimal residual CTLA-4 expression, which may still maintain partial immune homeostasis. Despite a homozygous genotype, clinical and immunological manifestations closely resemble those observed in individuals with heterozygous CTLA-4 insufficiency (15, 16, 17), thereby suggesting that CTLA-4 dosage critically influences the threshold between survival and immune dysregulation.

Patients possessing a homozygous *CTLA4* variant should be monitored in a manner consistent with that employed for individuals with LRBA deficiency and CTLA-4 insufficiency. Although the underlying molecular mechanisms differ, both conditions ultimately impair CTLA-4–mediated inhibitory signaling and lead to immune dysregulation (15, 16, 28). Consequently, clinical management should incorporate a multidisciplinary approach that encompasses immunological, rheumatological, and gastroenterological follow-up. Furthermore, biomarkers such as elevated cT_FH_ and T_H_1 responses, which decrease following abatacept therapy (21, 25, 28, 29, 30), may function as shared indicators for disease activity and therapeutic efficacy across these disorders.

The homozygous missense variant (S172P) disrupts CTLA-4 protein stability, resulting in enhanced lysosomal degradation. Treatment with the lysosomal inhibitors restored CTLA-4 expression, indicating that the variant accelerates lysosomal turnover rather than impairing synthesis. Mechanistically, this degradation pattern resembles the defective trafficking observed in LRBA-deficient cells (28, 31), suggesting that impaired protection of CTLA-4 from lysosomal degradation may represent a shared pathogenic mechanism.

Elevated expression of Helios and CD39 was observed in both T_con_ and Tregs in the patient, accompanied by increased Treg frequency and enhanced IL-10 production. Helios is a crucial protein for the stability and functional capacity of Tregs (32, 33). These findings suggest the activation of a compensatory immunoregulatory mechanism in response to decreased CTLA-4 expression, which may contribute to the relatively mild clinical phenotype observed. A similar phenotype has been documented in adult mice with Treg-specific deletion of CTLA-4, in which both FOXP3^+^ and FOXP3^-^ T cells exhibited elevated levels of IL-10 expression (34). Notably, the increased expression of Helios and CD39 in T_con_ cells may also reflect chronic T cell activation, consistent with reports indicating that CD39 is upregulated in exhausted T cells (35, 36).

In our patient, B cells exhibited a dysregulated phenotype characterized by an expansion of activated and atypical memory B cells, alongside a reduction in resting memory B cells. There was a significant increase in CD21^low^ CD38^low^ B cells, aligning with chronic immune activation and a shift toward extrafollicular and exhausted B cell phenotypes, as documented in systemic autoimmunity (37, 38, 39, 40, 41, 42, 43). The proliferation of DN2 B cells, which display extrafollicular characteristics, differentiate into plasma cells, and are closely related to activated naïve B cells, is observed in patients with systemic lupus erythematosus (43, 44). This profile suggests ongoing antigenic stimulation and compromised regulatory control over B cell activation. The simultaneous elevation of T_H_1-like T_FH_ cells indicates a skewed helper phenotype that correlates positively with the expansion of CD21^low^ B cells (45) but does not promote B cell survival or class switching (46). Overall, these findings imply that a pro-inflammatory environment fosters the expansion of atypical/exhausted B cells while depleting resting memory and naïve B cell populations, thereby contributing to immune dysregulation characteristic of chronic autoimmune states. Notably, abatacept treatment appeared to control chronic immune activation, accompanied by partial normalization of the B cell compartment, suggesting that restoring CTLA-4–mediated inhibition can help resolve persistent immune dysregulation.

In conclusion, the characterization of homozygous CTLA-4 deficiency provides the first comprehensive overview of its clinical and immunological spectrum. The condition presents as T cell hyperactivation combined with significant B cell dysregulation. These alterations indicate ongoing immune activation and disrupted regulatory control over adaptive immunity. Notably, treatment with abatacept effectively restored immune balance, normalizing both T and B cell phenotypes, and highlights the potential of targeted immunomodulatory therapy in managing CTLA-4–related immune dysregulation.

## Materials and methods

### Study design

A boy with a homozygous *CTLA4* variant was enrolled in this study. Ethical approval was obtained from Marmara University, and written informed consent was obtained from all participants. Baseline demographic, clinical, and immunological data were collected prospectively. Immunological assessments encompassed lymphocyte subset profiling, B and T cell subset analysis, cT_FH_ enumeration, intracellular cytokine analysis, recycling and intracellular CTLA-4 staining, CTLA-4 degradation assay with CHX, CTLA-4 rescue assay with MG132, and chloroquine treatment. To precisely capture the immunological impact of the treatment, changes in lymphocyte subsets (including Tregs, cT_FH_ cells, and B cell subtypes) and cytokine levels were evaluated at two strictly defined time points. Baseline evaluation was performed in the absence of systemic corticosteroids and immunomodulatory agents. The follow-up assessment was conducted after 12 mo of abatacept therapy, at which stage the patient was on abatacept monotherapy (supplemented only by supportive IVIG and antimicrobial prophylaxis).

### Antibodies and flow cytometry

Comprehensive multiparametric flow cytometry was performed to characterize lymphocyte populations, including T and B cell subsets, Tregs, and cT_FH_ cells. Detailed phenotypic profiling of CD4^+^ and CD8^+^ T cells was conducted to define T_H_1, T_H_2, and T_H_17 lineages, as well as naïve, central memory, effector memory, and terminally differentiated effector populations, and to evaluate CD31 expression. Intracellular cytokine staining was performed following in vitro stimulation to assess functional responses. The fluorochrome-conjugated monoclonal antibodies used for surface and intracellular staining are listed in Table S2. Peripheral blood lymphocyte subset analyses were performed by flow cytometry as described previously (25, 30). For lymphocyte subset analysis, 100 μl of whole blood or 5 × 10^5^ PBMCs were incubated with mAbs against surface markers for 20 min in the dark at room temperature. For whole-blood staining, red cells were lysed and washed before acquisition. For intracellular markers such as Helios, CTLA-4, and FOXP3, cells were fixed and permeabilized using eBioscience Foxp3/Transcription Factor Staining Buffer Set (Thermo Fisher Scientific), then stained with fluorochrome-conjugated specific antibodies for 1 h. For intracellular LRBA staining, heparinized whole blood was fixed and permeabilized using IntraPrep Permeabilization Reagent (Beckman Coulter). The samples were then incubated with a rabbit anti-human LRBA polyclonal primary antibody (1:100 dilution; Sigma-Aldrich) for 30 min at 4°C. Subsequently, the cells were stained with a secondary FITC-conjugated anti-rabbit IgG antibody (1:500 dilution; Thermo Fisher Scientific) for an additional 30 min at 4°C. For intracellular cytokine detection (IL-4, IL-17A, IL-10, and IFN-γ), cell suspensions (1 × 10^6^ cells) were stimulated with PMA (50 ng/ml) and ionomycin (1 μg/ml) in the presence of a protein transport inhibitor containing monensin and brefeldin A (BD Biosciences) for 5 h. After stimulation, cells were washed twice and stained for surface markers (CD4, CD8, and CD45RO). Fixed cell pellets were then permeabilized in a saponin-containing buffer (Thermo Fisher Scientific) and incubated with fluorochrome-conjugated, cytokine-specific antibodies for 45 min. All stained samples were acquired using a Navios EX flow cytometer (Beckman Coulter) and analyzed with FlowJo software (TreeStar).

### CD4^+^ T cell isolation, proliferation, and transendocytosis assay

CD4^+^ T cells were isolated from PBMCs using MojoSort Human CD4^+^ T Cell Isolation Kit (BioLegend) according to the manufacturer’s instructions. APC-Raji B cells (J159A; Promega) were resuspended in complete RPMI and treated with 20 µg/ml mitomycin C (BML-GR311; Enzo Life Sciences) at 37°C for 30 min. For the T cell proliferation assay, isolated CD4^+^ T cells were labeled with CellTrace Violet (Thermo Fisher Scientific). CD4^+^ T cells (2 × 10^5^) were cocultured with mitomycin C–treated APC-Raji B cells at a 2:1 ratio for 4 days. Following stimulation, cells were stained with APC-A700 CD4 (13B8.2, BC) and PC5.5 CD25 (B1.49.9, BC) and acquired on a Navios EX flow cytometer (Beckman Coulter).

To evaluate the transendocytosis capacity of CTLA-4, we utilized a CHO cell line stably expressing a CD80-mScarlet fusion protein. These CHO cells have previously been published (47) and were kindly provided by Prof. Bodo Grimbacher from Center for Chronic Immunodeficiency, University of Freiburg, Germany. Briefly, isolated CD4^+^ T cells were cocultured with the CD80-mScarlet–expressing CHO cells for 16 h under continuous stimulation with anti-CD2/CD3/CD28-coated beads (T Cell Activation/Expansion Kit, Miltenyi Biotec) in the presence of 30 µM chloroquine to prevent the lysosomal degradation of internalized CD80. Following co-incubation, the cells were harvested and surface-stained with APC-A700 anti-CD4 (clone 13B8.2; Beckman Coulter) and APC-A750 anti-CD45RA (clone 2H4; Beckman Coulter). The cells were then fixed and permeabilized using eBioscience Foxp3/Transcription Factor Staining Buffer Set (Thermo Fisher Scientific) according to the manufacturer’s protocol. Intracellular staining was subsequently performed using APC anti-CTLA-4 (clone BNI3; BioLegend) and eFluor 450 anti-FOXP3 (clone PCH101; Thermo Fisher Scientific) antibodies. Finally, the intracellular acquisition of CD80-mScarlet, along with the phenotypic markers, was acquired and quantified utilizing a CytoFLEX flow cytometer (Beckman Coulter).

### Cycling and intracellular CTLA-4 staining

PBMCs were stimulated with anti-CD3/anti-CD28 (1 μg/ml each) in 96-well plates for 3 days. Cells (2 × 10^5^) were stained with APC-A700 CD4 (13B8.2, BC) antibody and incubated for 1 h at 37°C and 5% CO_2_. For cycling staining, PE CTLA4 (BNI3, BC) was also added. After incubation, cells were fixed and permeabilized with eBioscience Foxp3/Transcription Factor Staining Buffer Set (Thermo Fisher Scientific). Alexa Fluor 647 FOXP3 (259D; BD Biosciences) and PE CTLA4 (BNI3, BC) were used for intracellular staining and incubated for 45 min. All stained cells were acquired using a Navios EX cytometer (Beckman Coulter) and analyzed by FlowJo software (TreeStar).

### CTLA-4 degradation and rescue assay

PBMCs were stimulated with anti-CD3 and anti-CD28 antibodies (each at 1 μg/ml) for 5 days, with 50 U/ml IL-2 added on day 3. To assess CTLA-4 protein degradation and estimate its half-life, cells were cultured at 37°C in 5% CO_2_ with CHX (30 μg/ml) (Sigma-Aldrich) for time points of 0, 2, and 4 h. For rescue experiments, cells were treated with chloroquine (50 μM) (Sigma-Aldrich) in the presence or absence of CHX, and with MG-132 (10 μM) (Sigma-Aldrich) in combination with CHX only, for 6 h. After treatment, cells were stained with Fixable Viability Dye eFluor 506 (Thermo Fisher Scientific) and APC-A700 CD4 (13B8.2, BC), followed by fixation and permeabilization using eBioscience Foxp3/Transcription Factor Staining Buffer Set (Thermo Fisher Scientific). Intracellular staining was conducted with Alexa Fluor 647 FOXP3 (259D; BD Biosciences) and PE CTLA-4 (BNI3, BC) for 45 min. CTLA-4 protein levels were measured in CD4^+^FOXP3^+^ cells using a Navios EX flow cytometer (Beckman Coulter).

### Immunoblot analysis

To assess LRBA and CTLA-4 expression in activated T cells, PBMCs activated with anti-CD3/CD28 for 4 days were lysed in an optimized buffer for 30 min on ice and cleared by centrifugation (15,000 rpm, 10 min, 4°C). Lysates were separated on a 4–15% Tris-glycine gradient gel and transferred to 0.45-µm polyvinylidene difluoride (PVDF) membranes. Membranes were blocked with 5% nonfat milk in phosphate-buffered saline with Tween 20 (PBS-T) and probed overnight at 4°C with rabbit anti-human LRBA (1:1,000; HPA023597; Sigma-Aldrich) and anti-CTLA-4 (clone F-8; Santa Cruz Biotechnology) primary antibodies. Following washes, blots were incubated with secondary antibodies for 1 h at room temperature and visualized using enhanced chemiluminescence.

To assess CTLA-4 effect on BiP expression, HEK293T cells overexpressing WT or S172P CTLA-4 were lysed, separated on 4–15% Tris-glycine gels, and transferred to PVDF membranes. Blots were probed with rabbit anti-DsRed (GTX59862; GeneTex) and rabbit anti-BiP (3177; Cell Signaling) antibodies.

### Generation of the CTLA4-DsRed-N1-S172P mutant construct

The c.514T>C mutation was introduced to the CTLA4-DsRed-N1 expression vector, kindly provided by Prof. Michael Lenardo (National Institutes of Health, Bethesda, MD, USA) (15) using the QuikChange Lightning Site–Directed Mutagenesis Kit (#210518; Agilent) following the manufacturer’s protocol. The following primer pair was used to introduce the mutation:

5′-AAA​ACA​ACC​CCG​GAC​TAA​CTG​CTG​CAA​GGA​TCC​AGA-3′.

5′- TCT​GGA​TCC​TTG​CAG​CAG​TTA​GTC​CGG​GGT​TGT​TTT-3′.

Colonies positive for the mutation were confirmed by Sanger sequencing using the following primers:

F1: 5′-CAT​CCC​TGT​CTT​CTG​CAA​AGC-3′.

R1: 5′-GCT​TTG​CAG​AAG​ACA​GGG​ATG-3′.

F2: 5′- CCA​ACA​GAG​CCA​GAA​TGT​G-3′.

R2: 5′-CCA​TCA​TGT​AGG​TTG​CCG-3′.

Two independently generated site-directed mutagenesis clones, each carrying the identical mutation, were used to perform overexpression experiments.

### CTLA-4 overexpression experiment

HEK239T cells (ATCC) were cultured in DMEM supplemented with 10% FBS and 100 U/ml penicillin/100 μg/ml streptomycin. TurboFect Transfection Reagent (#R0531; Thermo Fisher Scientific) was used to transiently transfect cells with 1 μg of CTLA4-DsRed-N1 or CTLA4-DsRed-N1-S172P. 48 h after transfection, cells were washed and surface CTLA4 was stained using APC-conjugated anti-CTLA4 antibody (#555855; BD Pharmingen). LIVE/DEAD Fixable Aqua Dead Cell Stain Kit was used to stain dead cells (#L34957; Thermo Fisher Scientific). Cells were then collected and run on ACEA NovoCyte (model 2010050) flow cytometer to assess DsRed expression and APC staining intensity. APC MFI was assessed on DsRed-positive cells and normalized to the levels from WT transfected cells in each independent experiment.

### WGS analysis

Genomic DNAs from patient and family members were whole genome–sequenced using the Illumina sequencing platform to an average depth of 30 reads per base in the Integrated Genomics Services Core Facility at Sidra Medicine. Reads were mapped to the human genome reference version hg19 using the Burrows–Wheeler Aligner. The Genome Analysis Toolkit (GATK) HaplotypeCaller was used for variant calling of single-nucleotide variants and indels. GATK Variant Quality Score Recalibration was used for variant quality filtering, and annotation was performed using SnpEff/SnpSift. Variants were prioritized based on quality scores, mode of inheritance, allele frequency below 0.01 in gnomAD, in silico deleteriousness predictions (CADD, SIFT, PolyPhen, MutationTaster), and relevance to the patient phenotype.

### Sanger sequencing

DNeasy Blood and Tissue Kit (#69504; Qiagen) was used to extract gDNA from PBMCs following the manufacturer’s instructions. Sanger sequencing was performed using the primer pair: 5′-GGC​TAC​CCA​TGC​AAT​TTA​GG-3′ and 5′-AAC​TCA​ACA​TCA​TCT​TTT​GGC​C-3′. Sequences were analyzed using Unipro UGENE software.

### Structural evolutionary analysis

The structural and evolutionary analysis of the identified variant was performed using a combination of sequence- and structure-based tools. The corresponding protein sequence was first analyzed by BLAST against the NCBI Reference Sequence protein database to identify homologous sequences. The resulting sequences were exported in FASTA format and aligned using Clustal Omega v1.2.4 (48, 49, 50). The multiple sequence alignment was visualized with ConSurf (51) to assess residue conservation. The predicted three-dimensional structure of the protein was obtained from the AlphaFold Protein Structure Database (52, 53). Structural visualization and analyses were performed using UCSF ChimeraX v1.10.1 (54).

### Statistical analysis

Data were expressed as the mean ± SD or median (minimum–maximum) based on distribution characteristics. One-way or two-way ANOVA with Tukey’s post hoc analysis and unpaired t tests were used for between-group comparisons, as appropriate. Statistical analyses were conducted using GraphPad Prism 10 (GraphPad Software Inc.).

### Online supplemental material

The provided supplemental materials present a comprehensive molecular and immunological characterization of the patient’s *CTLA4* variant, alongside their therapeutic response to abatacept. Genomic analyses, encompassing the WGS and Sanger sequencing data shown in Fig. S1 and Fig. S2, validate the familial segregation of the homozygous *CTLA4* variant in the index patient and heterozygous carriage in the parents. Additionally, Table S1 details other candidate variants identified via WGS. The functional consequences of this variant are demonstrated in Fig. S3 through comparative evaluations of CTLA-4 and LRBA protein expression levels among the patient, the heterozygous mother, and HCs. Clinically, Fig. S4 longitudinally maps the immunomodulatory efficacy of abatacept treatment by illustrating the pre- and posttreatment frequencies of Treg and naïve T cell subpopulations. Finally, the methodological framework underpinning these cellular analyses is established through the B cell immunophenotyping gating strategy outlined in Fig. S5 and the comprehensive inventory of utilized flow cytometry antibodies provided in Table S2.

## Ethical disclosure

The study protocol was approved by the local ethics committee of Marmara University, and written informed consent was obtained from all patients according to Good Clinical Practice guidelines.

## Supplementary Material

Table S1 lists other candidate variants identified in the index patient by WGS.

Table S2 lists antibodies used for flow cytometry.

SourceData F4 is the source file for Fig. 4.

SourceData FS3 is the source file for Fig. S3.

## Data Availability

The data generated during the study are included in this published article and its supplementary files.
