# Supplementary material for: A homozygous CTLA-4 variant causes CTLA-4 deficiency with severe immune dysregulation
Source: J Hum Immun. 2026 Jun 9;2(5):e20250227. doi: 10.70962/jhi.20250227 (PMC13248891; doi:10.70962/jhi.20250227)
Supplement: Table S1 — lists other candidate variants identified in the index patient by WGS. [file jhi_20250227_tables1.docx]

**Table S1.** List of other candidate variants identified in the index patient by WGS

| **Gene Names** | **Inheritance/Zygosity**^a^ | **PolyPhen** | **SIFT** | **Mutation Taster** | **Amino acid change** |
| --- | --- | --- | --- | --- | --- |
| PADI4 | Homozygous | possibly damaging | damaging | disease-causing | p.Arg650Ser |
| PROC | Homozygous |  |  | polymorphism | p.Ala142Val |
| PTPN18 | Homozygous | benign | damaging | polymorphism | p.Arg331Pro |
| CCDC150 | Homozygous | benign | tolerated | polymorphism | p.Ala657Ser |
| FZD5 | Homozygous | benign | tolerated | polymorphism | p.Arg211Gln |
| FEV | Homozygous | benign | tolerated | disease-causing | p.Gln129Arg |
| SPEG | Homozygous | benign | damaging | polymorphism | p.Ala2513Thr |
| LIFR | Homozygous | benign | tolerated | polymorphism | p.Asp816Gly |
| ATXN1 | Compound Heterozygous^b^ | possibly damaging | tolerated | disease-causing | p.Arg722Lys |
| ATXN1 | Compound Heterozygous^c^ |  |  |  | p.Gln219_Gln225del |
| SMPD2 | Homozygous | possibly damaging | damaging | polymorphism | p.Leu341Arg |
| NCOA7 | Homozygous | benign | damaging | polymorphism | p.Gly533Arg |
| TCF21 | Homozygous | benign | tolerated | polymorphism | p.Thr169Ala |
| SASH1 | Homozygous | possibly damaging | damaging | disease-causing | p.Pro487Leu |
| OGN | Homozygous | benign | tolerated | disease-causing | p.Ile249Val |
| KIAA1462 | Compound Heterozygous^b^ | benign | damaging | polymorphism | p.Gly1294Asp |
| KIAA1462 | Compound Heterozygous^c^ | benign | tolerated | polymorphism | p.Ser1007Asn |
| MUC19 | Homozygous |  | damaging |  | p.Arg271Cys |
| MUC19 | Homozygous |  |  |  | c.*5783-13387A>C |
| KMT2D | Homozygous | benign | tolerated | polymorphism | p.Phe372Tyr |
| KRT73 | Homozygous | benign | tolerated | polymorphism | p.Thr468Arg |
| KRT76 | Homozygous |  |  | disease-causing | p.Glu276Ter |
| RNF167 | Homozygous |  |  |  | p.Pro346ArgfsTer28 |
| HNF1B | Homozygous | possibly damaging | tolerated | disease-causing | p.Gln497Pro |
| KRT13 | Homozygous | benign | tolerated | polymorphism | p.Arg429His |
| HAP1 | Homozygous |  |  |  | p.Ala516_Ala522dup |
| ARSE | Hemizygous | possibly damaging | damaging | disease-causing | p.Met104Thr |
| CCDC22 | Hemizygous | possibly damaging | damaging | disease-causing | p.Asp440Tyr |
| IQSEC2 | Hemizygous | possibly damaging | damaging | disease-causing | p.Arg1345Trp |
| TMSB15B | Hemizygous |  |  |  | p.Val39Ala |
| UPF3B | Hemizygous | benign | tolerated | disease-causing | p.Thr149Ala |
| ABCD1 | Hemizygous | possibly damaging | damaging | disease-causing | p.Gly188Arg |
| MPP1 | Hemizygous | possibly damaging | tolerated | disease-causing | p.Ile375Thr |

Minor allele frequency <0.01.

^a^Variants that were homozygous in the patient listed above were heterozygous in both parents. Hemizygous variants were absent in the father and heterozygous in the mother.

^b^Inherited from heterozygous mother.

^c^Inherited from heterozygous father.
