# Supplementary material for: A homozygous CTLA-4 variant causes CTLA-4 deficiency with severe immune dysregulation
Source: J Hum Immun. 2026 Jun 9;2(5):e20250227. doi: 10.70962/jhi.20250227 (PMC13248891; doi:10.70962/jhi.20250227)
Supplement: Table S2 — lists antibodies used for flow cytometry. [file jhi_20250227_tables2.docx]

**Table S2.** List of antibodies used for flow cytometry

| Category | Marker | Fluorochrome | Clone | Manufacturer |
| --- | --- | --- | --- | --- |
| Lineage markers | CD45 | KO | J33 | Beckman Coulter |
|  | CD3 | FITC | UCHT1 | Beckman Coulter |
|  | CD3 | APC | UCHT1 | Beckman Coulter |
|  | CD4 | APC-A700 | 13B8.2 | Beckman Coulter |
|  | CD4 | APC-Cy7 | RPA-T4 | Biolegend |
|  | CD8 | PC7 | SFCI21Thy2D3 | Beckman Coulter |
|  | CD14 | APC-A700 | RMO52 | Beckman Coulter |
|  | CD16 | PE | 3G8 | Beckman Coulter |
|  | CD56 | PC5.5 | N901 | Beckman Coulter |
|  | CD19 | APC-A750 | J3-119 | Beckman Coulter |
| Differentiation / Memory markers | CD45RA | APC-A750 | 2H4DH11LDB9 | Beckman Coulter |
|  | CD45RA | FITC | HI100 | Biolegend |
|  | CD45RO | ECD | UCHL1 | Beckman Coulter |
|  | CCR7 (CD197) | PE | G043H7 | Beckman Coulter |
|  | CD31 | PB | 5.6E | Beckman Coulter |
| Regulatory T cells/ cT_FH_ cells/ Activation markers | CD25 | PC5.5 | B1.49.9 | Beckman Coulter |
|  | CD127 | APC-A750 | R34.34 | Beckman Coulter |
|  | CD39 | APC | BA54 | Beckman Coulter |
|  | CXCR5 (CD185) | APC | J252D4 | Beckman Coulter |
|  | PD-1 (CD279) | PE | PD1.3 | Beckman Coulter |
|  | CXCR3 | PE | G025H7 | Biolegend |
|  | CCR6 | PC7 | B-R35 | Beckman Coulter |
|  | FOXP3 | Alexa Fluor 647 | 259D | BD Biosciences |
|  | FOXP3 | eFluor™ 450 | PCH101 | Thermo Fisher Scientific |
|  | Helios | PB | 22F6 | Beckman Coulter |
|  | CTLA-4 | APC | BNI3 | Biolegend |
|  | CTLA-4 | PE | BNI3 | Beckman Coulter |
| B cell markers | CD20 | PB | B9E9 | Beckman Coulter |
|  | CD21 | PB | BL13 | Beckman Coulter |
|  | CD24 | ECD | ALB9 | Beckman Coulter |
|  | CD38 | PC5.5 | LS198-4-3 | Beckman Coulter |
|  | IgD | FITC | IA6-2 | Beckman Coulter |
|  | IgM | APC | SA-DA4 | Beckman Coulter |
|  | CD10 | APC-A700 | ALB1 | Beckman Coulter |
|  | CD11c | PC7 | BU15 | Beckman Coulter |
| Intracellular cytokines | IFN-γ | FITC | B27 | Biolegend |
|  | IL-4 | PC7 | MP4-25D2 | Biolegend |
|  | IL-10 | PE | JES3-9D7 | Biolegend |
|  | IL-17A | PerCP-Cy5.5 | BL168 | Biolegend |

**Abbreviations:** FITC, Fluorescein isothiocyanate; APC, Allophycocyanin; APC-A700, Allophycocyanin-Alexa Fluor 700; APC-A750, Allophycocyanin-Alexa Fluor 750; APC-Cy7, Allophycocyanin-Cyanin 7; KO, Krome Orange; PE, Phycoerythrin; PC7, Pycoerythrin-Cyanin 7; PC5.5, Pycoerythrin-Cyanin 5.5; PB, Pacific Blue; ECD, Phycoerythrin-Texas Red-x; AF647, Alexa Flour 647, Ig, immunoglobulin.
