# Supplementary figures and images for: A homozygous CTLA-4 variant causes CTLA-4 deficiency with severe immune dysregulation
Source: J Hum Immun. 2026 Jun 9;2(5):e20250227. doi: 10.70962/jhi.20250227 (PMC13248891; doi:10.70962/jhi.20250227)

Figure 4G

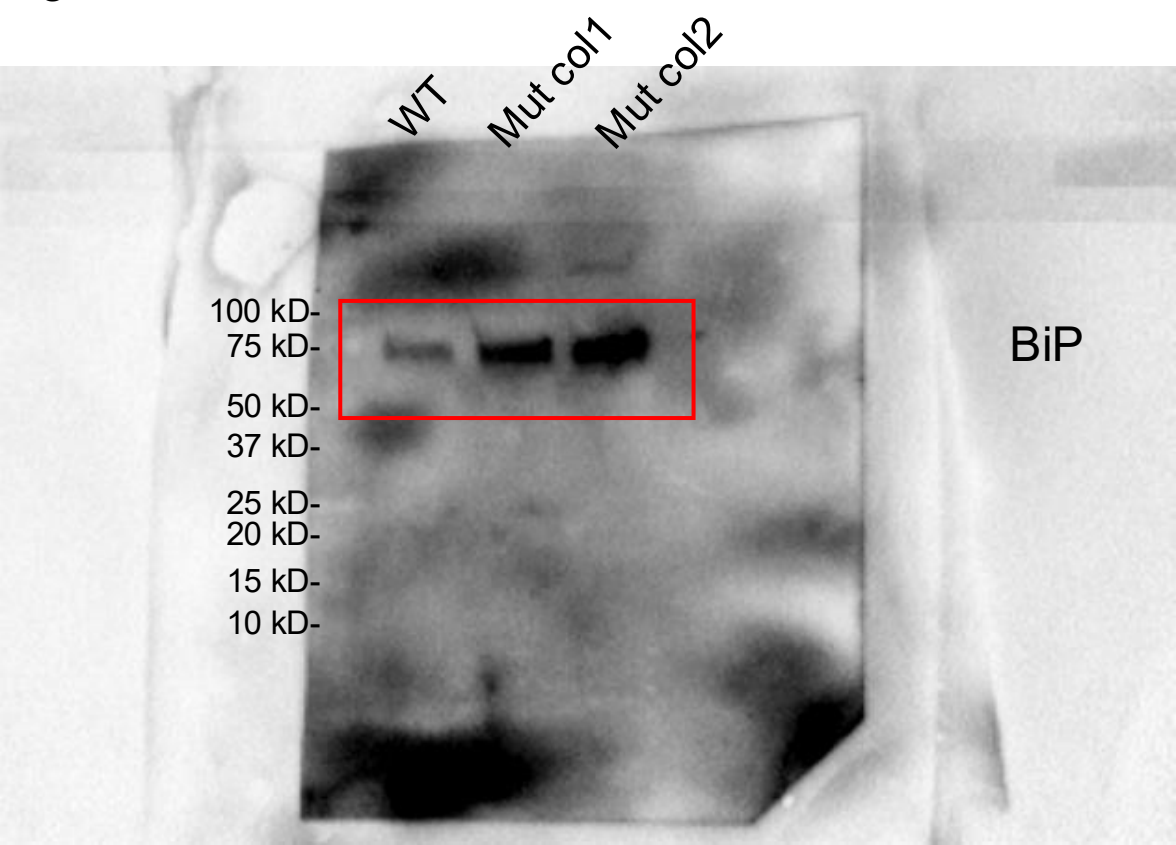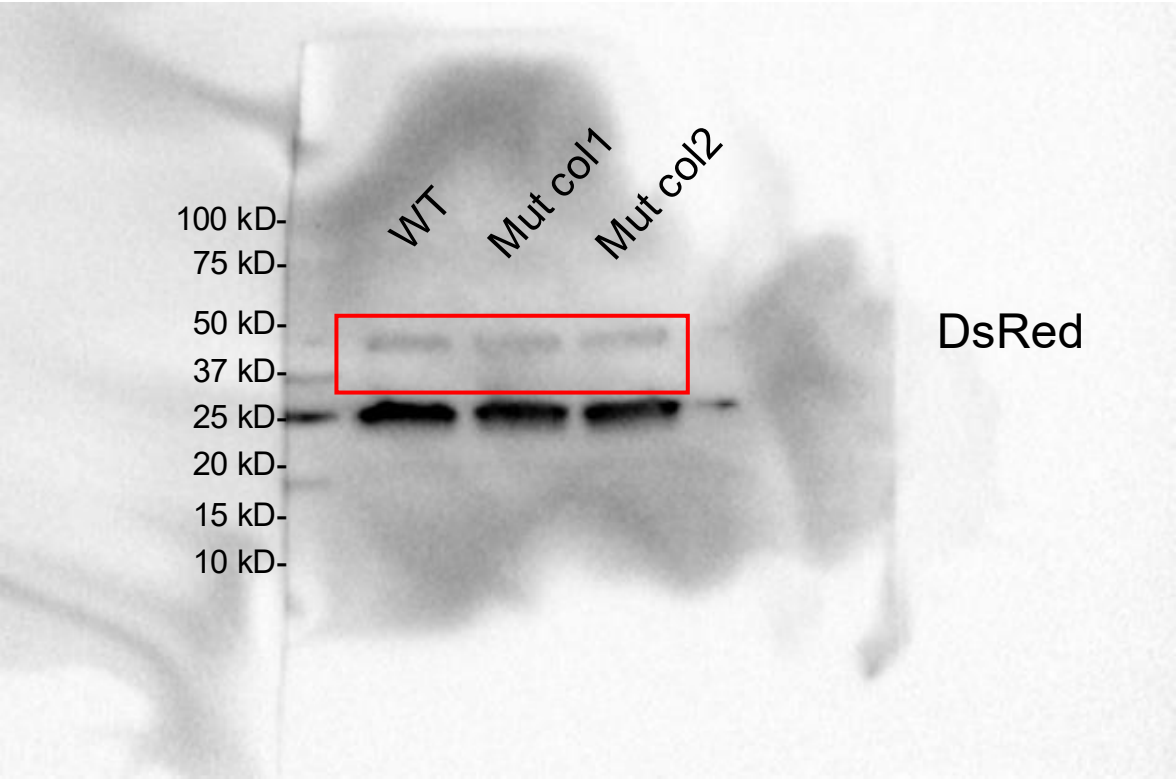

Figure 4G

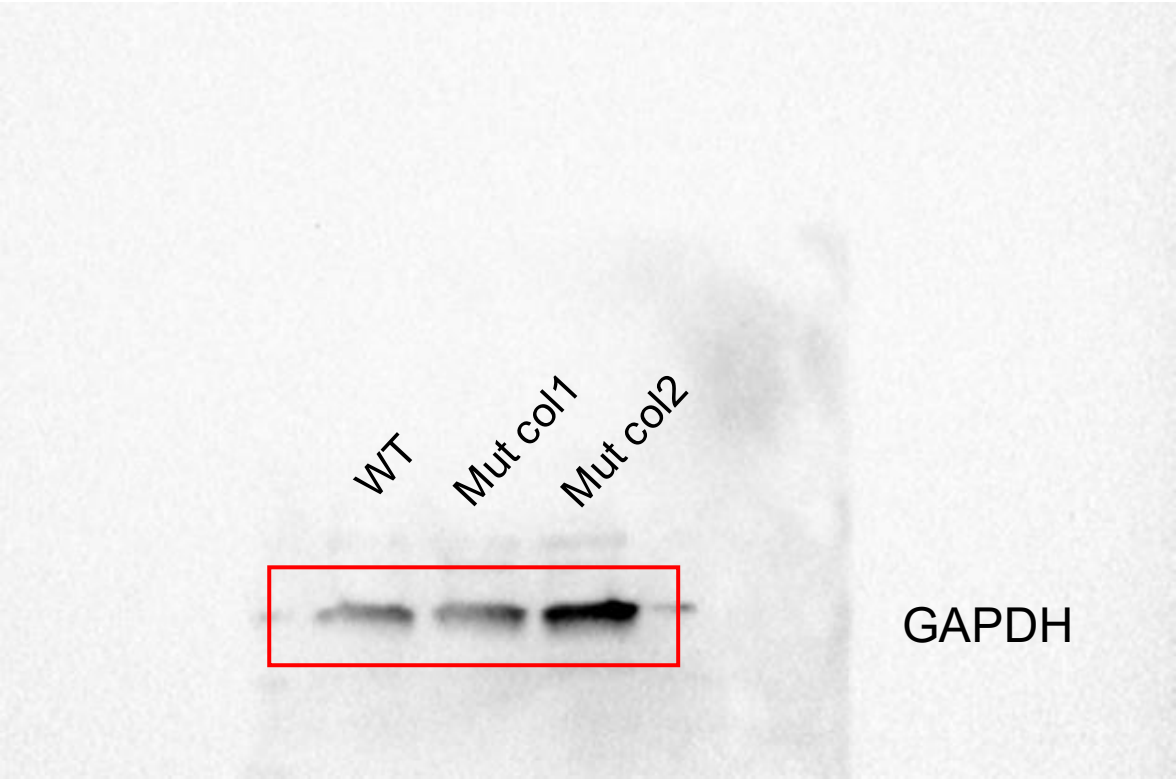

Supplement: SourceData F4 — is the source file for Fig. 4. [file jhi_20250227_sourcedataf4.pdf]

Figure S3A

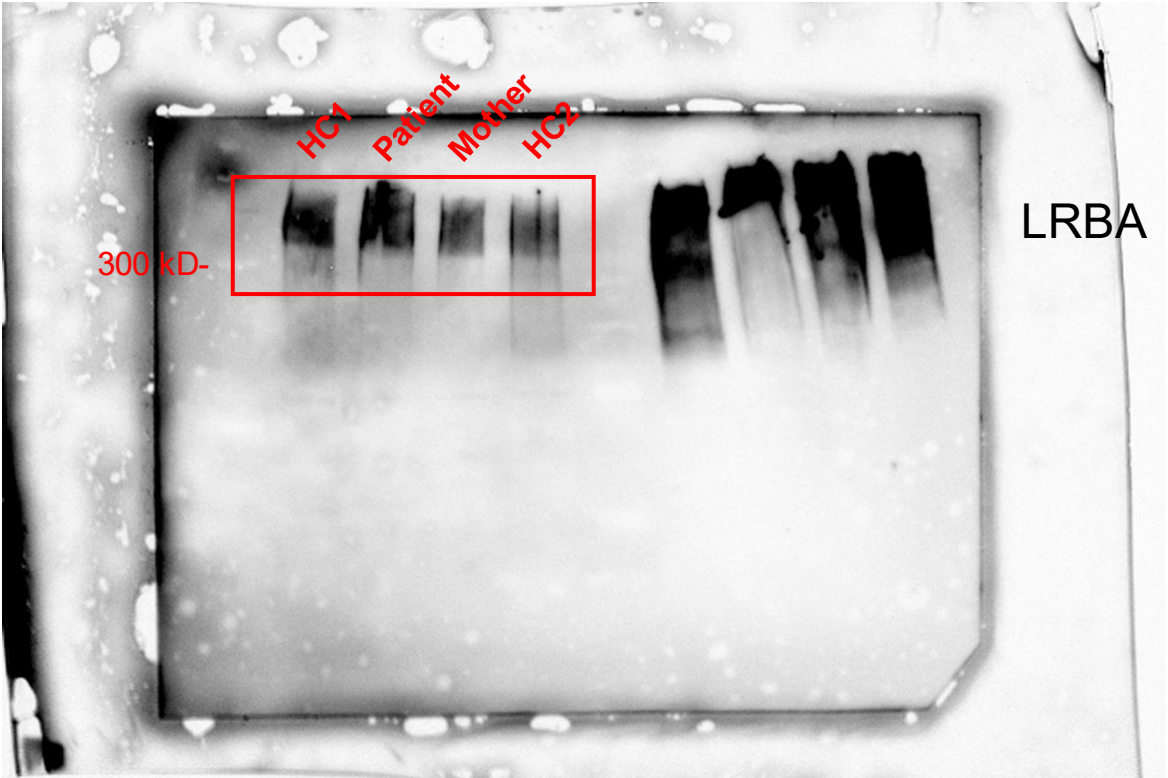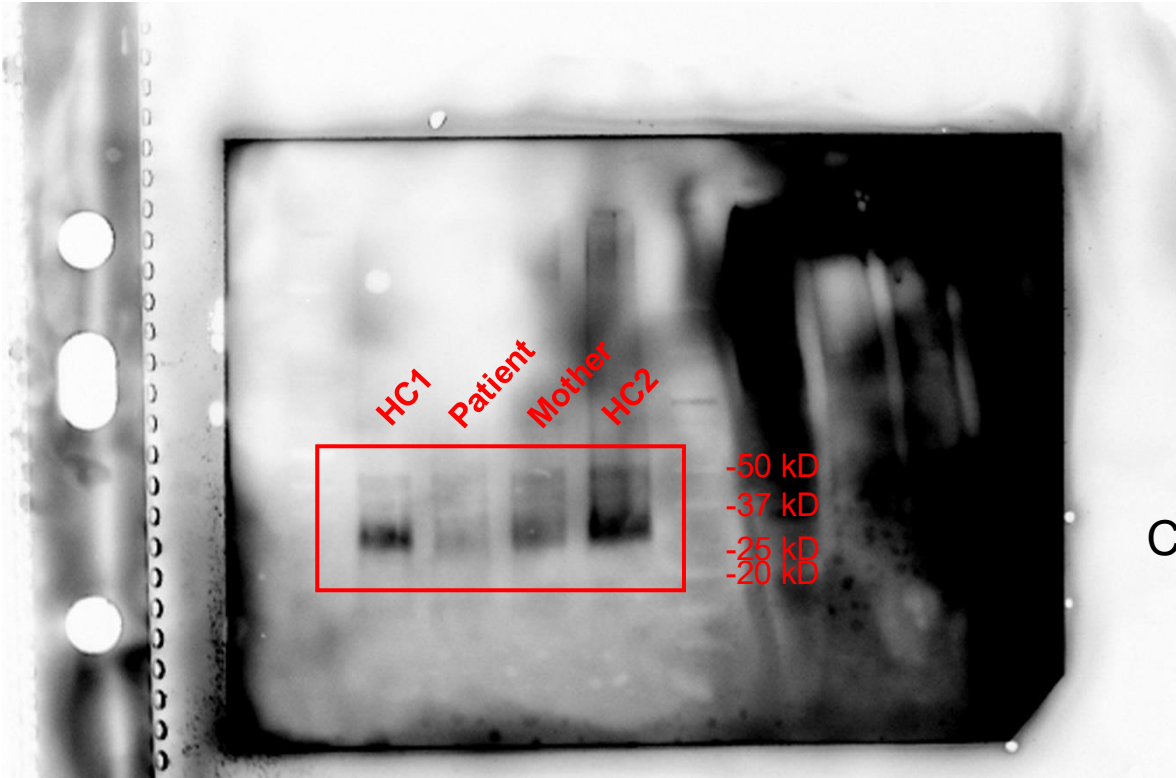

Figure S3A

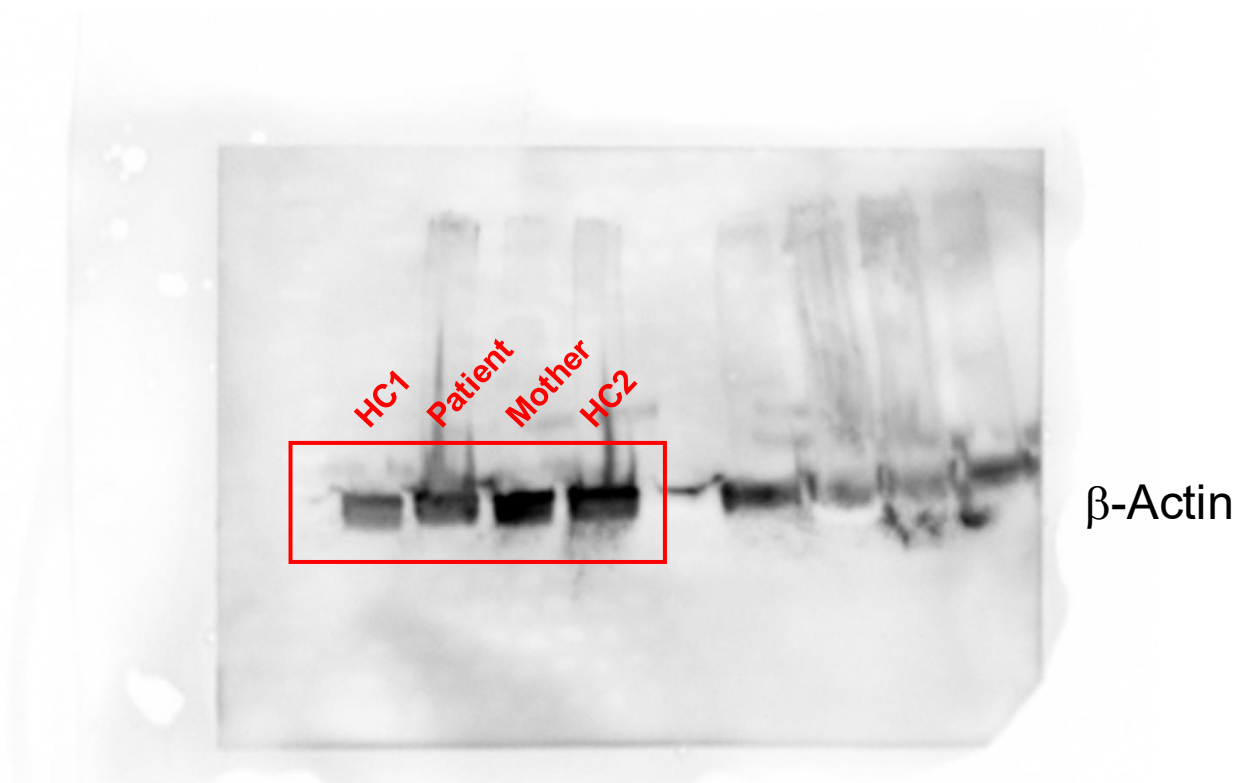

Supplement: SourceData FS3 — is the source file for Fig. S3. [file jhi_20250227_sourcedatafs3.pdf]
